# Single-cell transcriptomics reveals novel chondrocyte and osteoblast subtypes and their role in knee osteoarthritis pathogenesis

**DOI:** 10.1038/s41392-025-02136-8

**Published:** 2025-02-05

**Authors:** Yuan Liu, Wacili Da, Ming-Jie Xu, Chao-Xin Xiao, Tao Deng, Sheng-Liang Zhou, Xiao-Ting Chen, Yao-Jia Zhou, Li Tang, Yong Nie, Yi Zeng, Hui-Qi Xie, Bin Shen

**Affiliations:** 1 Department of Orthopedic Surgery and Orthopedic Research Institute, West China Hospital, Sichuan University, Chengdu, China; 2 Stem Cell and Tissue Engineering Research Center, State Key Laboratory of Biotherapy, West China Hospital, Sichuan University, Chengdu, China; 3 School of Mechanical Engineering, Sichuan University, Chengdu, China; 4 Animal Laboratory Center, West China Hospital, Sichuan University, Chengdu, China

**Keywords:** Experimental models of disease, Rheumatic diseases

## Abstract

Research on treating knee osteoarthritis (KOA) is becoming more challenging due to a growing number of younger patients being affected. The pathogenesis of KOA is complex for being a multifactorial disease affecting the entire joint, with remodeling of subchondral bone playing a key role in the degeneration of the overlying cartilage. Therefore, this study constructed a bipedal postmenopausal KOA mouse model to better understand how the interplay between subchondral bone remodeling and cartilage degeneration contributes to KOA development. A single-cell atlas of the osteochondral composite tissue was established. Furthermore, three novel subtypes of chondrocytes, including *Smoc2*^+^ angiogenic chondrocytes, *Angptl7*^+^ angiogenic chondrocytes, and *Col1a1*^+^ osteogenic chondrocytes, were identified in femoral condyles of KOA mice. In addition, the *Angptl7*^+^ chondrocytes promoted angiogenesis in the subchondral bone of KOA mice by interacting with endothelial cells via the FGF2-FGFR2 signaling pathway. The number of H-type vessels was increased in the subchondral bone, recruiting osteoprogenitor cells and facilitating osteogenesis in KOA mice. *Sparc*^+^ osteoblasts have negatively regulated bone mineralization and osteoblastic differentiation, aggravated the pathological remodeling of subchondral bone, and promoted the progression of KOA. The above findings have offered new targets and opened up an avenue for the therapeutic intervention of KOA.

## Introduction

Knee osteoarthritis (KOA) is one of the leading diseases impacting the quality of life of middle-aged and elderly individuals. It is increasingly being diagnosed at a younger age, with its onset now observed globally at a progressively earlier stage.^1^ This age shift in the disease’s onset has become a growing concern for public health worldwide. Pain, dysfunction, and even disability caused by the KOA require repeated and long-term treatment, resulting in substantial healthcare expenditures.^2^ In addition, the lifestyle changes brought about by the KOA may increase the chance of suffering from chronic diseases such as cardiovascular and cerebrovascular diseases, resulting in a great socioeconomic burden.^3^ Currently, there have been no effective pharmacological treatments that can completely alleviate the progression of KOA. However, the pathogenesis of KOA is complex for being a multifactorial disease affecting the entire joint.^4^ In particular, pathological remodeling of subchondral bone may be a key factor mediating the degeneration of the overlying cartilage. Osteoclasts are activated to promote the enhanced subchondral bone turnover and trabeculae deterioration at early stage of KOA.^5^ Although late-stage subchondral bone may exhibit sclerotic changes, elevated bone metabolism coupled with a reduced calcium-to-collagen ratio may result in inadequate bone mineralization.^6,7^ Therefore, when the mechanical load on the knee joint increases, stress-induced microfractures are likely to occur at the junction between the cartilage and the subchondral bone.^8^ The microfractures may lead to tidemark disruption, damaging the cartilage tissue barrier, promoting upward vascular invasion, and exacerbating cartilage degeneration.^9,10^ Given the significant role of subchondral bone remodeling in KOA development, elucidating its regulatory mechanisms may identify new therapeutic targets.

In recent years, the advent of single-cell RNA sequencing (sc-RNA-seq) technology has provided valuable new insights into the cellular heterogeneity and molecular mechanisms underlying the pathogenesis of KOA.^11,12^ This novel high-throughput sequencing approach has enabled researchers to explore the disease at a single-cell level, offering a detailed map of the cellular composition and the gene expression profiles of various cell types within affected tissues. Hu et al. have constructed a single-cell atlas for subchondral bones from tibial plateaus with the KOA and found that two novel endothelial cell populations and three osteoblast subtypes have been involved in the development of KOA.^13^ However, it may be insufficient to investigate subchondral bone remodeling without considering chondrocytes. The crosstalk between chondrocytes and subchondral bone cells plays a significant role in cartilage degeneration and subchondral bone remodeling during the progression of KOA.^14,15^ Additionally, as suggested by the histological evaluation of tibial plateaus of KOA patients, the blood vessels may invade the articular cartilage and promote degeneration at the early stage of KOA. Vascular granulation tissue and cartilage-like tissue were found deposited in the subchondral bone, becoming part of the bone remodeling process in late-stage KOA. Therefore, it is imperative to isolate tissues containing both cartilage and subchondral bone for clarifying the pathogenesis of subchondral bone remodeling in KOA through sc-RNA-seq and analysis.

The KOA mouse model has been selected for the sc-RNA-seq in order to mitigate the batch effects produced by waiting for normal control human specimens and minimize the confounding influence of comorbid conditions like diabetes on the subchondral bone microarchitecture of the KOA patients.^16,17^ However, there has been a lack of consensus on a KOA animal model that accurately mirrors the pathology of human disease.^18^ Currently, the majority of KOA animal models have been developed through post-traumatic and chemical injuries, but none has simulated the subchondral bone remodeling in KOA patients. The knee joint serves as a critical weight-bearing joint, and the decline in estrogen levels and the rise in body mass index (BMI) among elder women may initiate pathological alterations in the subchondral bone.^19,20^ Furthermore, postmenopausal women with high BMI have been the most common late-stage KOA patients.^21^ To address these issues, this study has developed a postmenopausal KOA model using bipedal mice, which were chosen to increase the mechanical load on the knee joints. This model was validated using finite element analysis, ensuring that the mechanical load on the knee joint accurately reflects the conditions seen in human KOA patients. Histological analysis at 22 weeks of age confirmed the presence of pronounced KOA phenotypes, including cartilage degeneration and bone changes similar to those observed in KOA patients.

This study presents a novel KOA mouse model characterized by estrogen withdrawal and increased mechanical load on the knee joints. Femoral condyles were isolated from the distal femur for sc-RNA-seq analysis, which allowed for the creation of a single-cell atlas of the femoral condyles in the KOA mouse model. By exploring the cellular heterogeneity and molecular mechanisms underlying the pathogenesis of KOA, the study uncovered important findings. Specifically, it was found that Angptl7+ chondrocytes and Sparc+ osteoblasts play key roles in mediating increased angiogenesis and abnormal bone remodeling, respectively. These findings suggest that targeting these cell populations may offer new therapeutic strategies for the treatment of KOA, providing hope for future advancements in the management of this debilitating disease.

## Results

### Pathological remodeling of subchondral bone in patients with KOA

**Fig. 1:**
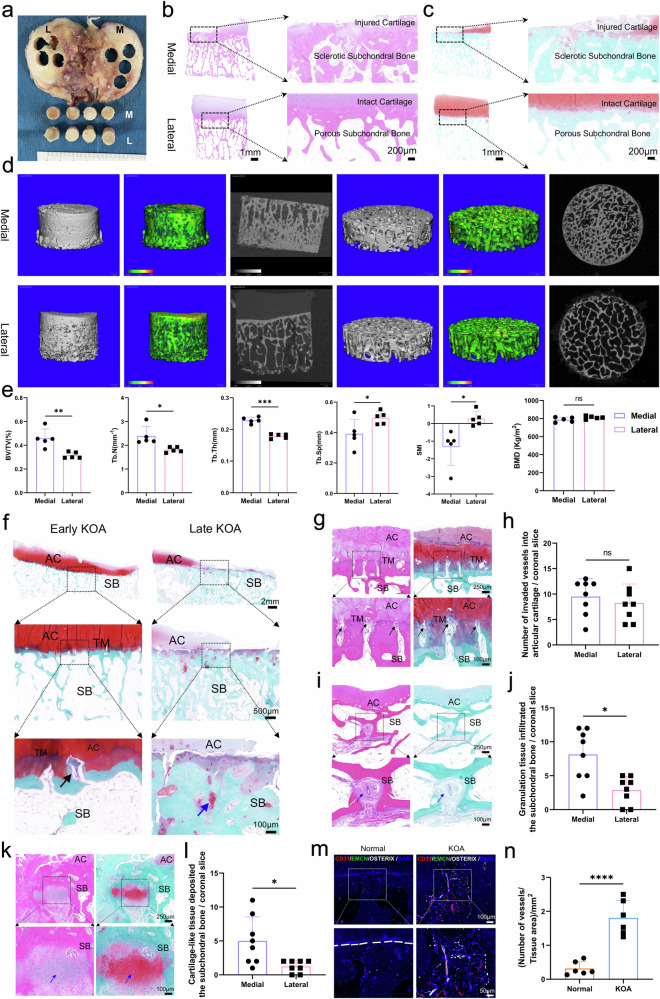
Pathological remodeling of subchondral bone in patients with KOA. **a** Tissue samples were isolated from tibial plateaus with KOA. **b**, **c** Representative sagittal micrographs of H&E (**b**) and SO&FG (**c**) staining for the samples of tibial plateaus with KOA, injured cartilage and subchondral bone lesions were marked. Scale bars = 1 mm, 200 μm. **d** Micro-CT measurement and heatmap of Tb.Th for the samples of tibial plateaus with the KOA. The color scale changes from green to red. The darker the color, the greater the Tb.Th. **e** Quantitative analysis of BV/TV, BMD, Tb.N, Tb.Th, Tb.Sp and SMI (*n* = 5 per group). The BMD of trabeculae on the medial tibial plateau is significantly lower than that on the lateral side. And the parameters measured in the lateral side appeared to be more concentrated compared with the medial side. **f** Representative sagittal micrographs of pathological alterations of tibial plateaus at early and late stage of KOA. Articular cartilage (AC), tidemark (TM), and subchondral bone (SB) are marked. Scale bars = 2 mm, 500 μm, 100 μm. **g** Representative micrographs of vascular invasion from the subchondral bone into articular cartilage. Scale bars = 250 μm, 100 μm. Black arrow indicates the vascular invasion into cartilage. **h** Quantitative analysis of invaded vessels crossing tidemarks in the medial and lateral tibial plateaus (*n* = 8 per group). **i** Representative micrographs of vascular rich granulation tissue infiltrated the subchondral bone with KOA. Scale bars = 250 μm, 100 μm. Scale bars = 100 μm, 50 μm. Blue arrow suggests the granulation tissue deposited in subchondral bone. **j** Quantitative analysis of the number of infiltrated granulation tissue in the medial and lateral subchondral bone (*n* = 8 per group). **k** Representative micrographs of cartilage-like tissue, positively stained with safranin O, deposited in the subchondral bone with KOA. Scale bars = 250 μm, 100 μm. Blue arrow suggests the cartilage-like tissue deposited in subchondral bone. **l** Quantitative analysis of the number of deposited cartilage-like tissue in the medial and lateral subchondral bone (*n* = 8 per group). **m** Representative micrographs of type H vessels (CD31^hi^EMCN^hi^) immune-positively stained with CD31 and EMCN in subchondral bone with KOA. White arrow indicates the type H vessels grown in subchondral bone with KOA. The white dashed line marked the boundary of the subchondral bone in the normal tibial plateau, while that was disrupted and unclear in the KOA group. **n** Quantitative analysis of the density of vessels in the normal and osteoarthritic tibial plateaus (*n* = 8 per group). Data are shown as mean ± SD. *P*-values were determined by paired, two-tailed *t*-test for (**e**, **h**, **j**, **l**) and unpaired, two-tailed *t*-test for (**n**)

By following the instructions from the OARSI and ICRS,^22^ tibial plateau samples were collected from patients with primary KOA who underwent knee replacement surgery (Supplementary Table 1, Supplementary Fig. 1). Cylindrical specimens with a diameter of 8 mm were drilled from both the medial and lateral tibial plateaus (Fig. 1a). For KOA patients with varus deformity, H&E (Fig. 1b) and SO&FG (Fig. 1c) staining showed more severe progression of KOA in the medial tissue of the tibial plateau compared with the lateral side. The lateral cartilage tissue showed less damage and even distribution of trabeculae. In contrast, the medial cartilage tissue wore away, and there was a noticeable and regional subchondral bone hyperplasia and sclerosis. Micro-CT examination further proved that the trabecular bone in the medial tibial plateaus of the KOA patients was significantly denser compared with the lateral side (Fig. 1d). Additionally, compared to the subchondral bone microstructure of the lateral tibial plateau, the medial trabeculae have a significantly greater BV/TV (*p* = 0.006), number (*p* = 0.020) and thickness (*p* = 0.0001), and lower separation (*p* = 0.026) and SMI (*p* = 0.021), but reduced bone mineral density (MD = −23.90 Kg/m^2^, *p* = 0.232) (Fig. 1e). This suggests that in KOA, the more severe subchondral bone sclerosis remodeling of the medial tibial plateau is accompanied by insufficient bone mineralization.

Further histological analysis revealed that, in the early stages of KOA, vessels invaded the articular cartilage, and in the later stages, cartilage-like tissue was deposited within the subchondral trabeculae (Fig. 1f). Vascular invasion was accompanied by the destruction of tidemark, thickening of calcified cartilage, and degeneration of hyaline cartilage (Fig. 1g). And the number of vessels invaded into cartilage was not significantly different between medial and lateral tibial plateaus (Fig. 1h). The infiltration of vascular-rich granulation tissue within the trabecular bone (Fig. 1i) along with the deposition of cartilage-like tissue positive for safranin O (Fig. 1k), are important pathological features of subchondral bone remodeling. These phenomena were markedly more prevalent and pronounced in the medial tibial plateaus of individuals with KOA, where the pathological alterations were considerably more severe compared to the lateral side (Fig. 1j, l). Type H vessels, a newly identified subtype of bone vasculature characterized by endothelial cells with high expression of CD31 and EMCN, have been found to be closely associated with osteogenesis.^23^ Through multiple immunofluorescence staining using CD31 and EMCN antibodies, it has been confirmed that Type H vessels are indeed present in the subchondral bone of individuals with KOA (Fig. 1m). Furthermore, the density of these vessels within a given area was significantly higher in the KOA group compared to the normal control group (Fig. 1n). Consequently, it can be inferred that the increased angiogenesis, deposited cartilage-like tissue and the activated bone remodeling processes are markedly joining and promoting the development of KOA disease.

### Postmenopausal KOA model is successfully constructed in bipedal mice

**Fig. 2:**
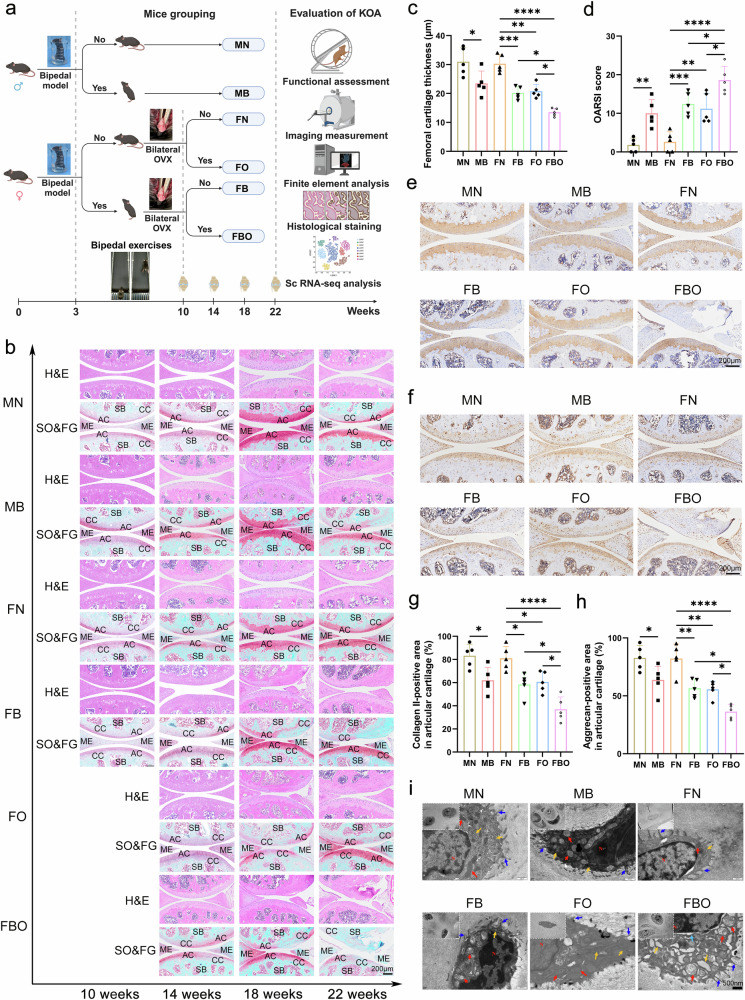
Postmenopausal KOA model was successfully constructed in bipedal mice. **a** A schematic of mice experimental design. This study has included six groups of mice: MN (male normal mice), MB (male bipedal mice), FN (female normal mice), FB (female bipedal mice), FO (female mice with bilateral ovariectomy), and FBO (female bipedal mice with bilateral ovariectomy). The forelimbs and tail of 3-week-old C57 mice were cut off in a sterile environment, and exercise for the bipedal activities was performed on a treadmill with a slow speed. Half of female mice were bilaterally ovariectomized at 10 weeks of age. The phenotype of KOA was evaluated monthly from 10 to 22 weeks of age. **b** Representative micrographs of H&E and SO&FG staining in sagittal sections of the medial sides of knee joints of experimental mice at 10, 14, 18, and 22 weeks of age. Articular cartilage (AC), calcified cartilage (CC), subchondral bone (SB) and meniscus (ME) are marked. Scale bar = 200 μm. **c** Measurement and quantitative analysis of femoral cartilage thickness (*n* = 5 per group). **d** The Osteoarthritis Research Society International score for different animal models (*n* = 5 per group). **e**, **f** Representative micrographs of immunohistochemical staining of Collagen II (**e**) and Aggrecan (**f**) in sagittal sections of the medial sides of the knee joints in the six groups at 22 weeks of age, Scale bar = 200 μm. **g**, **h** Semi-quantitative analysis of immune-positive percentage of Collagen II (**g**) and Aggrecan (**h**) (*n* = 5 per group). **i** Representative TEM images of the chondrocytes in cartilage tissues of knees at 22 weeks of age, Scale bars = 2 μm, 500 nm. Red, orange, cyan and blue arrows indicated the mitochondrial, rough endoplasmic reticulum, perinuclear space, and cell protrusions, respectively. Data are shown as mean ± SD. *P*-values were determined by one-way ANOVA with a Tukey post hoc test for (**c**, **d**, **g**, **h**)

The mouse grouping and experimental setup are shown in Fig. 2a. This study included a total of six groups of mice: MN (male normal mice), MB (male bipedal mice), FN (female normal mice), FB (female bipedal mice), FO (female mice with bilateral ovariectomy), and FBO (female bipedal mice with bilateral ovariectomy). The forelimbs and tails of C57 mice at 3 weeks of age were amputated, with half of the female mice undergoing bilateral ovariectomy at 10 weeks of age. The KOA phenotype was assessed monthly from 10 to 22 weeks of age.

Compared to the other five groups of mice at the ages of 10, 14, and 18 weeks, H&E and SO&FG staining revealed that, in the FBO group, the degenerative changes in articular cartilage had progressively worsened, with cartilage tissue being eroded and deformed by the age of 22 weeks (Fig. 2b). The thickness of femoral cartilage was significantly decreased (Fig. 2c), and the OARSI score was significantly increased (Fig. 2d). The loss of main components of the extracellular matrix (ECM) of chondrocytes in the FBO group mice, including collagen II (Fig. 2e, g) and aggrecan (Fig. 2f, h), was most pronounced at 22 weeks of age. Furthermore, transmission electron microscopy (TEM) observation of cartilage tissue in the FBO group showed damaged chondrocytes with swollen mitochondria, expanded rough endoplasmic reticulum, and widened perinuclear space (Fig. 2i).

**Fig. 3:**
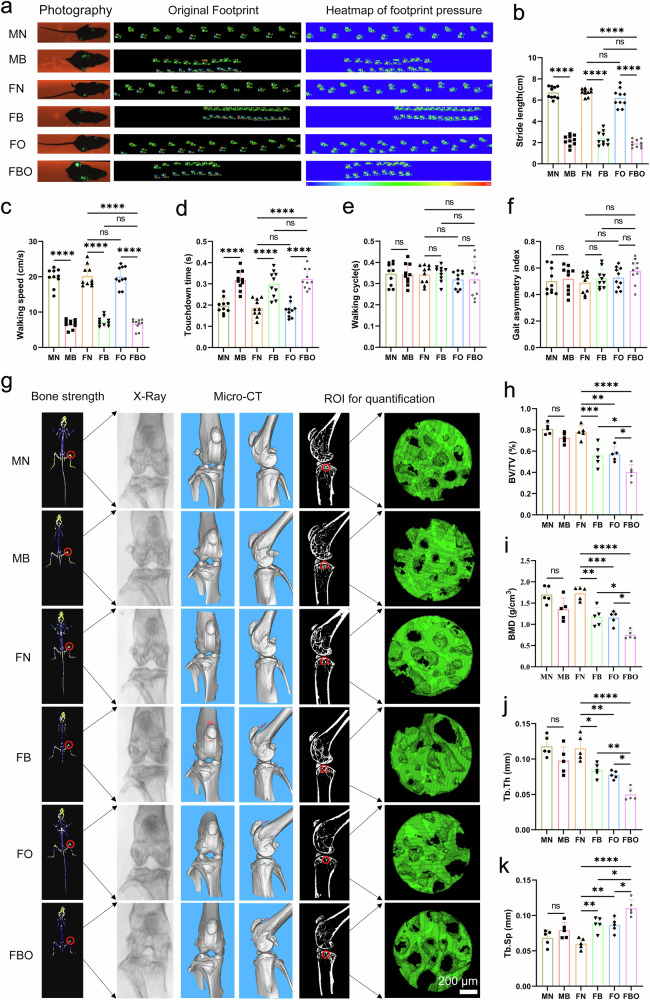
Gait analysis and micro-CT measurement of KOA mice. **a** Results of gait analysis of mice at 22 weeks of age, including the original footprint and heatmap of footprint pressure. **b** Quantitative analysis of stride length (*n* = 5 per group). **c** Quantitative analysis of walking speed (*n* = 5 per group). **d** Quantitative analysis of touchdown time (*n* = 5 per group). **e** Quantitative analysis of walking cycle (*n* = 5 per group). **f** Quantitative analysis of gait asymmetry index (*n* = 5 per group). **g** Representative images of bone strength heatmap and micro-CT images for the 3-dimensional (3D) reconstruction of knee joints at 22 weeks of age. Scale bar = 200 μm. **h** Quantitative analysis of BV/TV (*n* = 5 per group). **i** Quantitative analysis of BMD (*n* = 5 per group). **j** Quantitative analysis of Tb.Th (*n* = 5 per group). **k** Quantitative analysis of Tb.Sp (*n* = 5 per group). Data are shown as mean ± SD. *P*-values were determined by one-way ANOVA with a Tukey post hoc test for (**b**–**f** and **h**–**k**)

There were significant differences in gait between the bipedal mice and normal mice (Fig. 3a), despite the bipedal mice being able to move freely within their cages. Compared with normal mice, the bipedal mice exhibited markedly shortened stride length (Fig. 3b), slower walking speed (Fig. 3c), longer touchdown time (Fig. 3d), but similar walking cycle (Fig. 3e) and gait asymmetry index (Fig. 3f). The imaging examination revealed an obvious decrease in bone strength in the hindlimbs of FBO mice, along with narrowing of the knee joint space, an increased presence of osteophytes, and varus deformity when compared to FN mice (Fig. 3g). In addition, subchondral bone damage with decreased BV/TV, BMD, and Tb.Th, and increased Tb.Sp was found in the FBO mice compared to FN mice (Fig. 3h–k). Taken together, the above findings suggested that the KOA phenotypes were most pronounced in FBO mice at 22 weeks of age.

### Pathological remodeling of subchondral bone in KOA mice

**Fig. 4:**
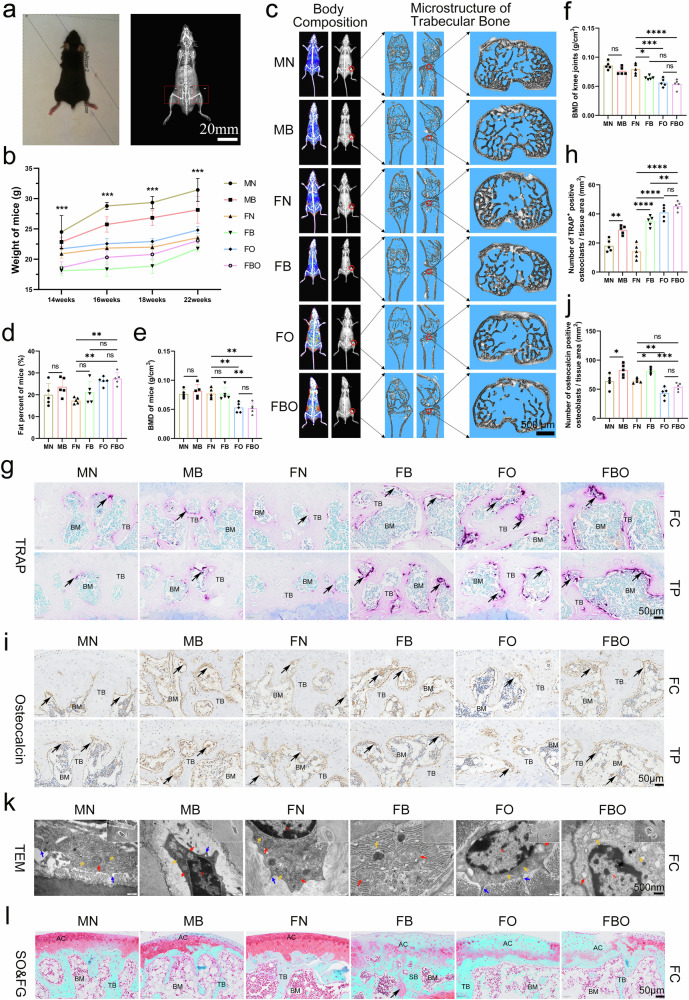
Pathological remodeling of the subchondral bone in the KOA mice. **a** The BMD and body composition (bone mineral content, fat mass, and lean mass) of the mice were measured by Inalyzer (left), and the corresponding X-ray photograph of mice (right). Red box indicated the selected region of interest of knee joints for analysis. Scale bar = 20 mm. **b** Changing trend and quantitative analysis of weights of mice in six groups from 14 to 22 weeks of age (*n* = 5 per group). **c** Representative images of body composition heatmap and microstructure of trabecular bone of mice in six groups at 22 weeks of age. The bone is white, fat is red, blue & green are mixed lean and liquid. Scale bar = 500 μm. **d**, **e** Quantitative analysis of percentage of fat (**d**) and BMD (**e**) of mice in six groups at 22 weeks of age (*n* = 5 per group). **f** Quantitative analysis of the BMD of the knee joints of mice in six groups at 22 weeks of age (*n* = 5 per group). **g**, **h** Representative sagittal micrographs of TRAP staining (**g**) and related semi-quantitative analysis (**h**) in subchondral bone of femoral condyles (FC) and tibial plateaus (TP) of mice in the six groups at 22 weeks of age (*n* = 5 per group). Scale bar = 50 μm. Black arrows indicated the TRAP^+^ osteoclasts. **i**, **j** Representative sagittal micrographs of IHC staining of osteocalcin (**i**) and related semi-quantitative analysis (**j**) in subchondral bone of mice in the six groups at 22 weeks of age (*n* = 5 per group). Scale bar = 50 μm. Black arrows indicated the TRAP^+^ osteoclasts or osteocalcin^+^ osteoblasts. **k** Representative TEM images of osteoblasts in subchondral bone of femoral condyles of mice in the six groups at 22 weeks of age. Scale bars = 2 μm, 500 nm. Red, orange and blue arrows indicated the mitochondrial, rough endoplasmic reticulum, and cell protrusions, respectively. **l** Representative sagittal micrographs of SO&FG staining of femoral condyle (FC) of mice in the six groups at 22 weeks of age, indicating the cartilage degeneration and pathological remodeling of subchondral bones. Articular cartilage (AC), subchondral bone (SB) and bone marrow cavity (BM) were marked. Scale bar = 50 μm. Black arrow indicated the cartilage-like tissue deposited in the subchondral bone. Data are shown as mean ± SD. *P*-values were determined by one-way ANOVA with a Tukey post hoc test for (**b**, **d**, **e**, **f**, **h**, and **j**)

The BMD and body composition (bone mineral content, fat mass, and lean mass) of the mice in six groups were assessed using the InAlyzer (Fig. 4a). From the age of 14 to 22 weeks, the weight of the bipedal mice was significantly less than that of the normal mice. When they grew to 18 and 22 weeks of age, the weight of the FO mice increased a lot and was significantly higher than that of the FN mice. Furthermore, the weight of the FBO mice increased much more than that of FB mice (Fig. 4b). In addition, the percentage of fat of FO and FBO mice at 22 weeks of age was significantly increased than that of the FN mice (Fig. 4c, d), but the BMD of FO and FBO mice was significantly lower (Fig. 4c, e). The damage to the subchondral bone microstructure of FBO mice was the most pronounced among the six groups (Fig. 4c). The BMD of the knee joint of MN mice was the highest among all groups, and it was significantly lower in FO and FBO mice than that of FN mice (Fig. 4f). That is, compared with normal mice, model mice exhibited a higher proportion of fat deposition surrounding the knee joint and a lower proportion of lean tissue.

Specifically, the quantity of TRAP^+^ osteoclasts showed a significant increase in the subchondral bone of FB, FO, and FBO mice (Fig. 4g, h). Additionally, the number of osteocalcin^+^ osteoblasts significantly increased in the subchondral bone of FB and FBO mice (Fig. 4i, j). These findings suggest that both subchondral bone resorption and formation were notably activated in FBO mice. Furthermore, observation of osteoblasts in the femoral condyle using TEM showed swollen mitochondria of osteoblasts in MB, FB, and FBO mice, and expanded rough endoplasmic reticulum of osteoblasts in FBO mice, potentially impacting protein synthesis (Fig. 4k). Notably, histological changes similar to the pathological remodeling of subchondral bone in KOA patients were also observed in FB and FBO mice, such as vascular invasion and cartilage-like tissue deposition (Fig. 4l).

### Single-cell transcriptional landscape of femoral condyles in KOA mice

**Fig. 5:**
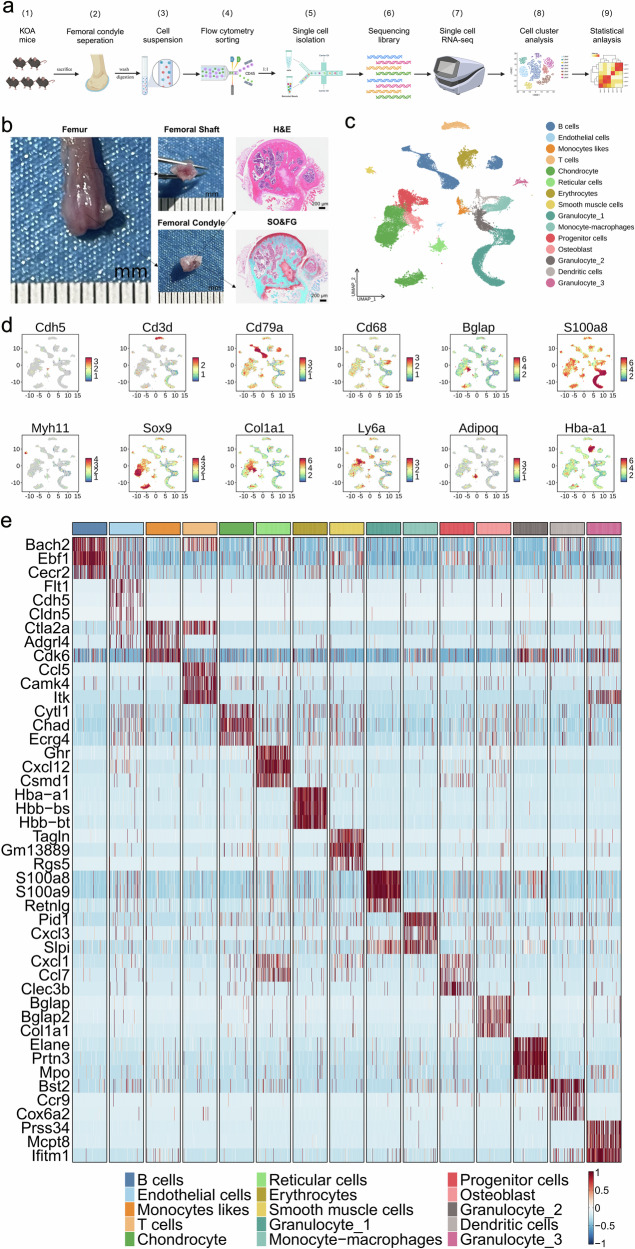
The single-cell landscape of femoral condyles in six groups at 22 weeks of age. **a** Schematic workflow for transcriptomic profiling of the mice femoral condyles with KOA using scRNA-seq. The scRNA-seq data (one sample per group, six groups) were assayed by following the SeekOne protocol, including 5 pairs of knees in each sample. The CD45^+^ cells were selected by flow cytometric sorting, and the CD45^+^ and CD45^−^ cells were mixed in a ratio of 1:1 before the examination. **b** Process of isolation for femoral condyle from femur for scRNA-seq, and representative images of isolated femoral condyles stained with H&E and SO-FG, Scale bar = 200 μm. **c** UMAP plot of single cells profiled in the presenting work colored by cell types. A total of 65,491 cells were isolated from six groups of femoral condyles and divided into 15 cell types. **d** Feature plots showing the expression of key markers in various clusters projected on the UMAP plot. Red indicates high expression and blue indicates low or no expression. **e** Heatmap revealing the scaled expression of the top 3 discriminative genes in each cell cluster defined in (**c**), the color scheme is based on z-scores

To define cell populations and identify genome-wide gene expression patterns in the development of subchondral bone remodeling and cartilage degeneration. Ten femoral condyles from hindlimbs of 5 mice in each group at 22 weeks of age were isolated for scRNA-seq using a modified STRT strategy^24^ (Fig. 5a). Femoral condyle used for scRNA-seq was isolated manually at the junction connected the femoral shaft and condyle, to eliminate the influence of chondrocytes distributed in the growth plate (Fig. 5b). A total of 82,083 cells were isolated from femoral condyles in mice of six groups, and 65,491 cells were retained for subsequent analysis after rigorous filtration (Supplementary Table 2). That consisted of 26 clusters (Supplementary Fig. 2a), which were identified as 15 cell types (Fig. 5c), including chondrocytes (CC, 16,378), endothelial cells (EC, 553), osteoblast (OB, 2203), progenitor cells (PC, 5461), reticular cells (RC, 2184), B cells (11,463), T cells (2528), monocytes likes (1193), monocyte-macrophages (4404), dendritic cells (1155), erythrocytes (2929), smooth muscle cells (819), granulocyte-1 (10,657), granulocyte-2 (2821), granulocyte-3 (743) (Supplementary Fig. 2b–d). Identity of each cell type was determined based on the expression of previously published specific marker genes (Fig. 5d, e, and Supplementary Fig. 2e). For example, CC was identified for high expression of *Sox9*, *Acan*, and *Col2a1*; EC was defined for high expression of *Pecam1*, *Cldn5*, and *Cdh5*; OB was confirmed for high expression of *Alpl*, *Bglap*, *Col1a1*, and *Ibsp*; PC was identified for high expression of *Cd34*, *Ly6a*, and *Thy1*; RC was defined for high expression of *Adipoq*, *Cxcl14*, and *Gdpd2*. Additionally, the volcano plots of differentially expressed genes in all cell types across the different groups were shown in Supplementary Figs. 3–8.

### Enhanced mechanical stress accelerates cartilage degeneration

**Fig. 6:**
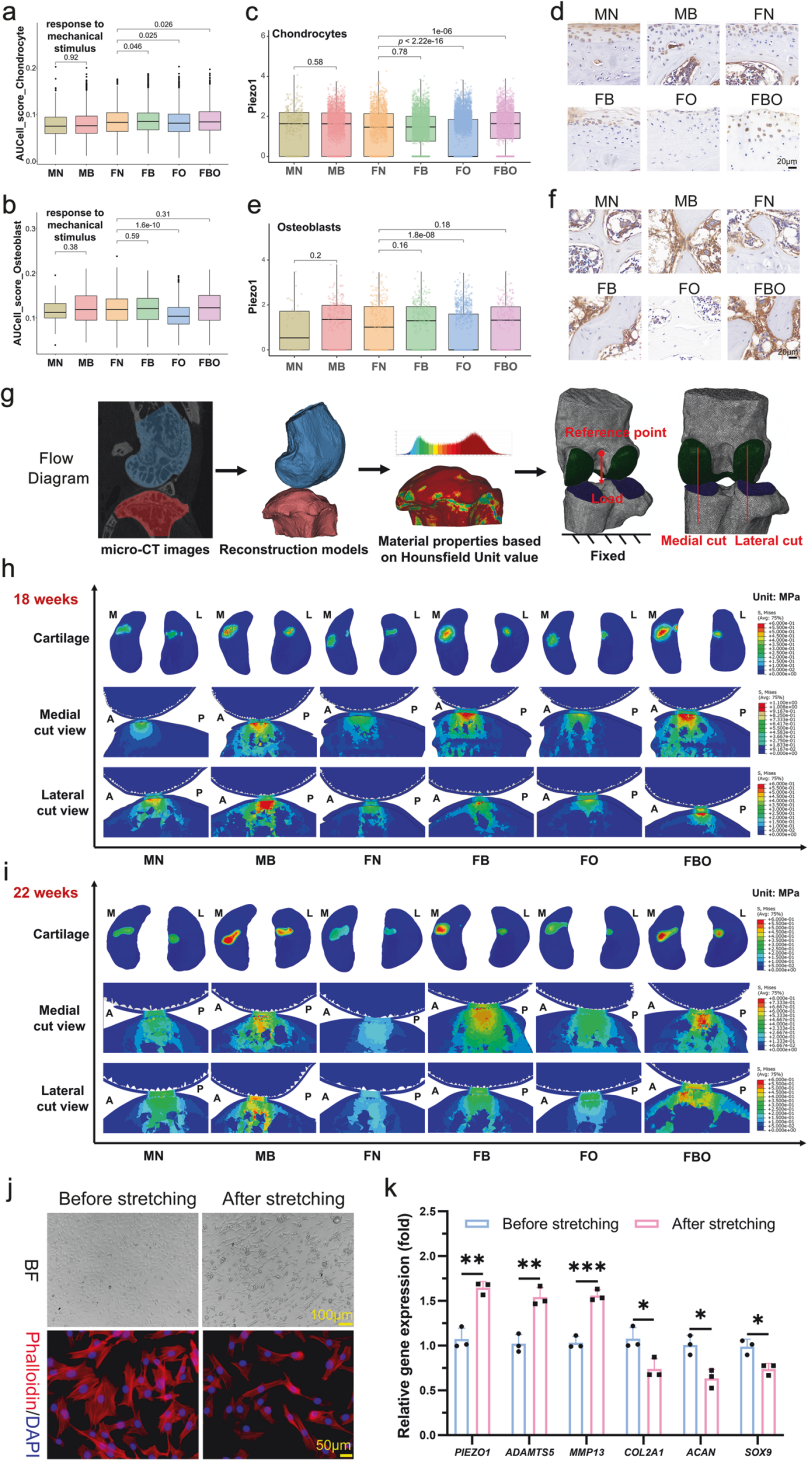
Enhanced mechanical stress accelerated cartilage degeneration. **a**, **b** The box plot represents the scores of chondrocytes (**a**) and osteoblasts (**b**) in each group for “response to mechanical stimulus” pathway in the GO database, scores come from the R package “AUcell”. **c**, **d** The box plot represents the *Piezo1* gene expression of chondrocytes (**c**), and representative images of immunohistochemistry staining of indicated *Piezo1* gene in cartilage tissue (**d**) in each group, Scale bar = 20 μm. **e**, **f** The box plot represents the *Piezo1* gene expression of osteoblasts (**e**), and representative images of immunohistochemistry staining of indicated *Piezo1* gene in subchondral bone (**f**) in each group, Scale bar = 20 μm. **g** Schematic representation of the workflow followed for Finite Element Analysis (FEA) of the models reconstructed from DICOM data of knees measured by micro-CT. **h**, **i** Heatmap of stress distribution (MPa) on cartilage surface and subchondral bone of knee joints in six groups at 18 (**h**) and 22 (**i**) weeks of age. The stress distribution is mainly concentrated on the medial side of the knee joints, and the stress intensity in bipedal mice was significantly higher than that in normal and FO mice. **j** Representative images of chondrocytes in bright field or staining with phalloidin (red) to present the cytoskeleton changes of chondrocytes before and after stretching for 24 h, Scale bars = 100 μm, 50 μm. **k** Expression level of genes related to mechanical stress and cartilage degeneration, including *PIEZO1*, *ADAMTS5*, *MMP13*, *COL2A1*, *ACAN* and *SOX9*, of chondrocytes before and after stretching for 24 h. Data are shown as mean ± SD, *n* = 3 per group. *P*-values were determined by unpaired, two-tailed *t*-test

AUCell package was used to score the “response to mechanical stimulus” pathways of CCs and OBs of samples from six groups. In comparison to the FN group, the AUCell scores were significantly higher in the FB and FBO groups, and significantly lower in the FO group (Fig. 6a, b). The changes in expression levels of *Piezo1*, a mechanically-activated ion channel that links mechanical forces to biological signals, exhibited consistent patterns with AUCell scores in both CCs (Fig. 6c) and OBs (Fig. 6e), as confirmed by IHC staining (Fig. 6d, f). Furthermore, the finite element analysis was performed for the mechanical stress distribution on the cartilage surface and subchondral bone based on the constructed three-dimensional models of knee joints of mice in each group (Fig. 6g). Bipedal mice at 18 and 22 weeks of age had higher mechanical stress in cartilage and subchondral bone compared to normal and FO mice, with FBO mice having the highest stress levels (Fig. 6h, i). These findings indicated that the study effectively increased the stress intensity of the knee joint using the bipedal mouse model.

In order to further explore the effect of mechanical stress on CCs, this study constructs an in vitro CC stress stimulation model using the IonOptix C-Pace EM stretch system. After 24 h of stretching, it was observed that CCs exhibited a notable elongation and narrowing of their shape and skeletal structure (Fig. 6j). Moreover, the expression levels of *PIEZO1* gene and ECM-degrading metalloproteinases such as *ADAMTS5* and *MMP13* of CCs significantly increased after stretching, while the expression levels of ECM synthesis-related genes such as *COL2A1*, *ACAN* and *SOX9* significantly decreased (Fig. 6k). The aforementioned findings suggest that enhanced mechanical stress may contribute to cartilage degeneration by reducing ECM synthesis.

### Aggravated chondrocyte damage, vessel invasion and subchondral bone remodeling in the KOA mice

**Fig. 7:**
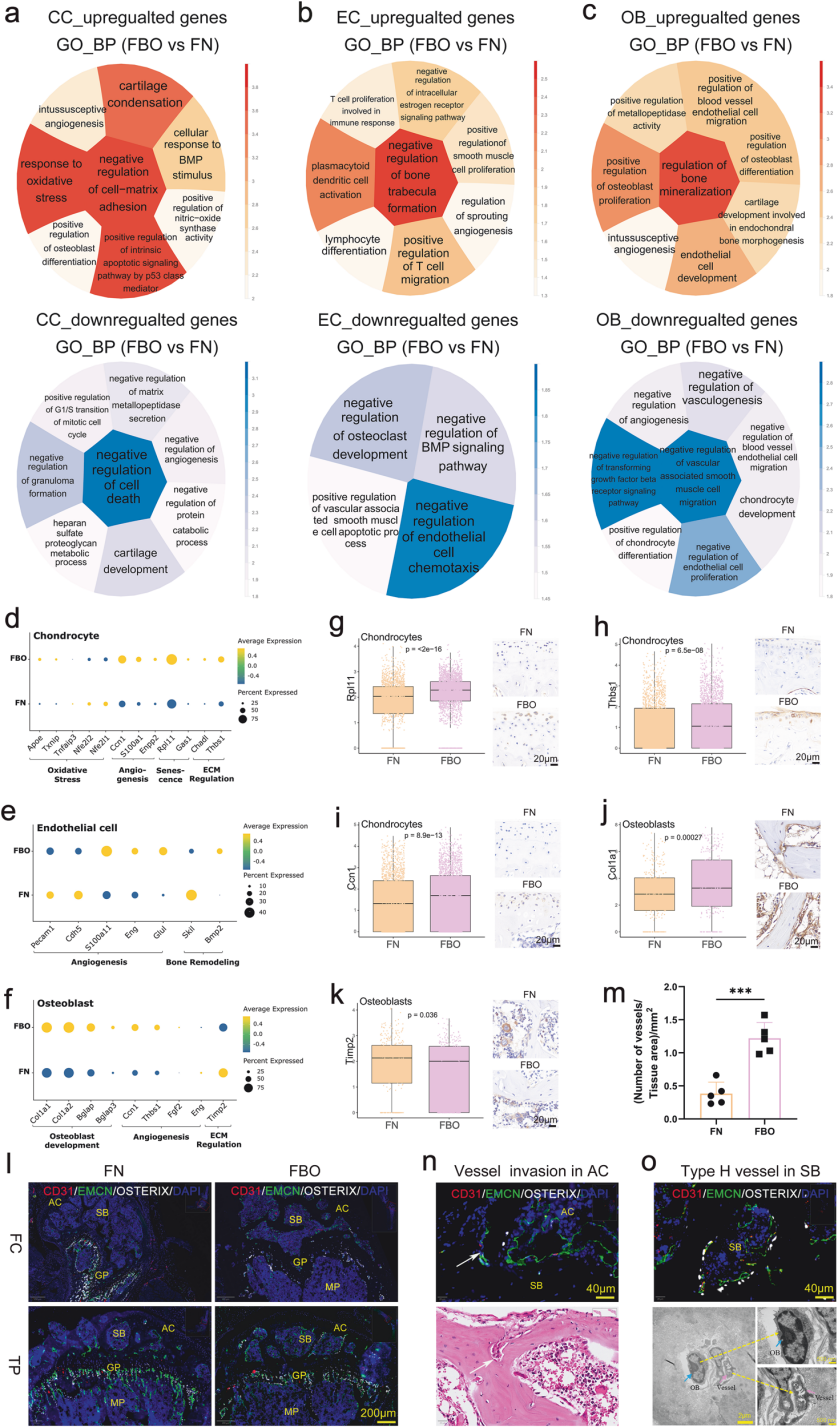
Chondrocyte damage, vessel invasion and subchondral bone remodeling was aggravated in the KOA mice. **a**–**c** The circle enrichment plot shows the GO database results of group FBO compared with group FN in chondrocytes (**a**), endothelial cells (**b**) and osteoblasts (**c**). The deeper the color or the bigger the size is, the −log_10_(*p*-value) is greater, indicating a more significant difference. Red (YlOrRd) circle plot indicated the up-regulation function of group FBO relative to group FN (left), and the blue (PuBu) circle plot indicated the down-regulation function (right). **d**–**f** Dot plots indicated differentially expressed genes of chondrocytes (**d**), endothelial cells (**e**) and osteoblasts (**f**) between the FBO and FN groups. Presented genes are derived from the results of interest in the enrichment analysis and mostly refer to degeneration of chondrocytes, angiogenesis and remodeling of subchondral bone. **g** The box plot represents the *Rpl11* gene expression of chondrocytes related to the “senescence” pathway in (**d**). **h** The box plot represents the *Thbs1* gene expression of chondrocytes related to the “ECM regulation” pathway in (**d**). **i** The box plot represents the *Ccn1* gene expression of chondrocytes related to the “angiogenesis” pathway in (**d**). **j** The box plot represents the *Col1a1* gene expression of osteoblasts related to the “osteoblast development” pathway in (**f**). **k** The box plot represents the *Timp2* gene expression of osteoblasts related to the “ECM regulation” pathway in (**f**). All referred genes were confirmed in vivo by IHC staining, Scale bar = 20 μm. **l** Representative images of multi-immunofluorescence staining for type H vessels (CD31^hi^EMCN^hi^) grown in femoral condyles (FC) and tibial plateaus (TP) of FN and FBO mice. Articular cartilage (AC), subchondral bone (SB), growth plate (GP), and metaphysis (MP) were marked. *Osterix*^+^ progenitor cells (white) distributed a lot in the growth plate of FN mice, while it significantly decreased in FBO mice. And vessels highly expressed CD31 (red) and EMCN (green), even *osterix*^+^ progenitor cells (white) appeal around it, grown more in the subchondral bone of FBO mice than FN. Scale bar = 200 μm. **m** Quantitative analysis of the density of vessels in the subchondral bone of knees from the FN and FBO mice. Data are shown as mean ± SD, *n* = 5 per group. *P*-values were determined by unpaired, two-tailed *t*-test. **n** Representative images of multi-immunofluorescence and H&E staining for CD31^hi^EMCN^hi^ vessels (white arrow indicated) invaded into articular cartilage of FBO mice. Scale bar = 40 μm. **o** Representative images of multi-immunofluorescence staining for type H vessels grown in SB of FBO mice. Scale bar = 40 μm. And representative TEM images of osteoblast grown around the vessel. Scale bars = 2 μm, 500 nm

The FB mice have shown increased osteoclast differentiation and inhibited BMP signaling compared with the FN mice, while the MB mice showed an opposite trend compared with the MN mice (Supplementary Figs. 9–11). This could explain why the KOA phenotype of FB mice progressed more rapidly than that of MB mice during the same observation period. Compared to FN mice, alterations of gene expression of CCs in FBO mice were significantly related to oxidative stress, angiogenesis, senescence, and ECM dysregulation (Fig. 7a, d). Changes in the gene expression of the ECs in the FBO mice regulated abnormal vascular proliferation and pathological remodeling of the subchondral bone (Fig. 7b, e). Altered gene expression of the OBs in the FBO mice has enhanced osteoblast development, angiogenesis, and ECM degradation (Fig. 7c, f). Among these, the expression level of aging-related gene *Rpl11* was significantly increased in the CCs of FBO group compared with that in the FN group (Fig. 7g). And so was *Thbs1*, a gene associated with the dysregulation of ECM (Fig. 7h). Angiogenic gene *Ccn1* was upregulated in CCs of FBO mice than that of FN mice (Fig. 7i). The expression level of *Col1a1*, relevant to OB differentiation, was significantly increased in the OBs of FBO group compared with that in the FN group (Fig. 7j). *Timp2*, a gene inhibited the expression level of matrix metalloproteinase, was significantly downregulated in the OBs of FBO mice compared with that of FN mice (Fig. 7k).

The potential results concerning the alterations in gene expression in the CCs, ECs, and OBs in the FBO sample were validated. It was found that the blood vessels have significantly proliferated in the subchondral bone (Fig. 7l, m), while *Osterix*^+^ osteoprogenitor cells and blood vessels were obviously reduced in the growth plate of the FBO mice compared with the FN mice. Further observation revealed that the blood vessels have invaded the articular cartilage of the FBO mice (Fig. 7n). And CD31^hi^ EMCN^hi^ H-type vessels, recruiting *Osterix*^+^ osteoprogenitor cells, have grown in the subchondral bone of the FBO mice, which was closely coupled with osteogenesis (Fig. 7o). In addition, the expression of TRAP (Fig. 4g, h) in the subchondral bone of the FBO mice was significantly increased compared with the FN mice, which indicated the pathological remodeling of subchondral bones.

### *Angptl7*^+^ chondrocytes promote angiogenesis through the FGF2-FGFR2 signaling pathway in the KOA mice

**Fig. 8:**
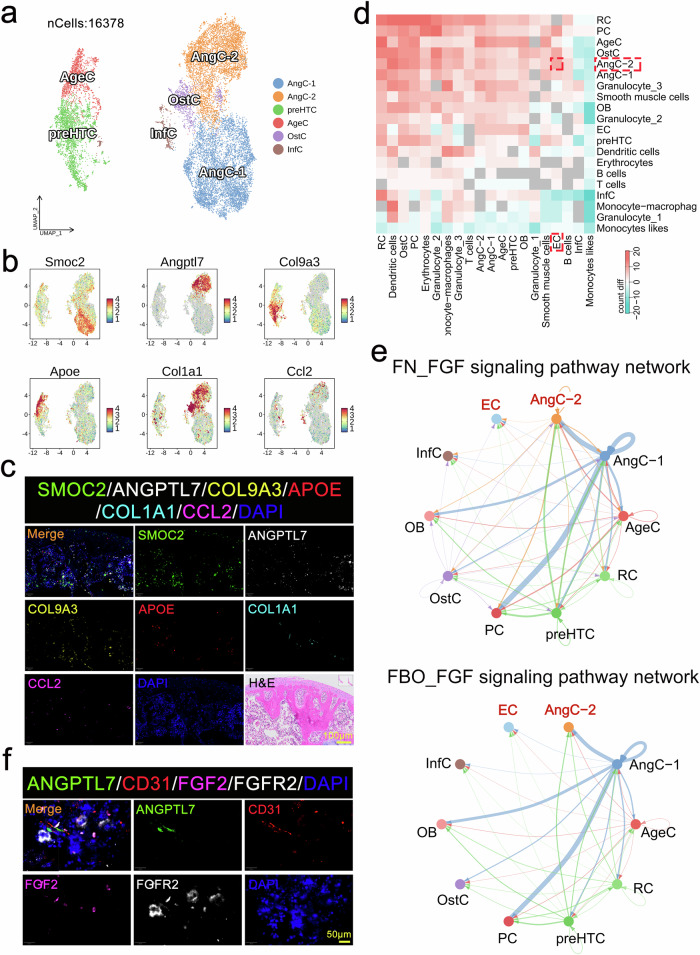
*Angptl7*^+^ chondrocytes promoted angiogenesis through the FGF2-FGFR2 signaling pathway in the KOA mice. **a** Six sub cell types of 16,378 chondrocytes were visualized by a UMAP plot, including angiogenic chondrocytes-1 (AngC-1, 6263), angiogenic chondrocytes-2 (AngC-2, 4800), prehypertrophic chondrocytes (preHTC, 2651), aging chondrocytes (AgeC, 1362), osteogenic chondrocytes (OstC, 939), inflammatory chondrocytes (InfC, 363). **b** Expression patterns of selected markers projected on the UMAP plot. Red color indicated high expression and blue color indicated low or no expression, one marker for each sub cell type of chondrocytes was shown. **c** Representative images of immunofluorescence and H&E staining for marking the location and expression of six subtypes of chondrocytes in a FBO sample. Scale bar = 50 μm. **d** The heatmap shows the number of interactions in FBO group and FN group among each cell sub cell types. Red indicates a higher number of interactions in group FBO; blue indicates a higher number of interactions in group FN. **e** Circle plot showing the inferred FGF signaling networks in FN and FBO datasets. Edge line thickness indicated the interaction strength of FGF signaling between different cell types. The interaction between AngC-2 and EC was significantly improved through FGF signaling pathway in FBO sample. **f** Representative images of multi-immunofluorescence staining for the interaction between AngC-2 and EC through the FGF2-FGFR2 signaling pathway in the FBO sample. Scale bar = 50 μm

Six sub cell types of 16,378 chondrocytes of six samples were visualized by a UMAP plot (Supplementary Fig. 16a), including angiogenic chondrocytes-1 (AngC-1, 6263), angiogenic chondrocytes-2 (AngC-2, 4800), prehypertrophic chondrocytes (preHTC, 2651), aging chondrocytes (AgeC, 1362), osteogenic chondrocytes (OstC, 939), inflammatory chondrocytes (InfC, 363) (Fig. 8a, Supplementary Fig. 16b, c). Based on the GO enrichment analysis of genes highly expressed in each subtype of CCs (Fig. 8b, Supplementary Fig. 16d–i), it was found that both AngC-1 and AngC-2 had the function of promoting angiogenesis (Supplementary Fig. 16d, e), and highly expressed *Smoc2* and *Angptl7*, respectively; PreHTC highly expressed *Col9a3*, which was closely related to CCs hypertrophy (Supplementary Fig. 16f); AgeC highly expressed *Apoe*, that was closely associated with senescence (Supplementary Fig. 16g); OstC highly expressed *Col1a1* and other related genes, that was closely coupled with osteoblast differentiation and bone development (Supplementary Fig. 16h); InfC highly expressed *Ccl2*, which was closely correlated with inflammation (Supplementary Fig. 16i). The number and position of six subpopulations of CCs in the femoral condyle of FBO mice were presented through multiplex IF staining of marker genes (Fig. 8c).

It was likely that AngC-2 is involved in the abnormal proliferation of vessels in the subchondral bone of FBO mice, rather than AngC-1 (Supplementary Fig. 17a–d). Compared with the FN sample, the expression of *Cebpb*, *Cst3*, *Ecrg4*, *Thbs1*, *Ubb*, *Rpl11*, and *S100a10* in AngC-2 cells of the FBO sample was significantly upregulated, while the expression of *Cdk8*, *Hk2*, *Fn1*, *Bmp2*, and *Nfkb1* was significantly downregulated (Supplementary Fig. 17f). Cell interaction analysis revealed that the interaction between AngC-2 and ECs in the FBO mice was obviously enhanced compared with that in the FN mice (Fig. 8d). And pathway screening reveals that AngC-2 may enhance its regulatory effects on ECs through the FGF signaling pathway (Fig. 8e and Supplementary Fig. 18). Further analysis found that AngC-2 in the FBO mice was believed to interact with ECs through the FGF2-FGFR2 ligand-receptor pair, which was also observed by multiplex IF staining (Fig. 8f and Supplementary Fig. 19).

To further investigate the role of *ANGPTL7*^+^ chondrocytes in cartilage degeneration and KOA progression, we constructed human chondrocyte models with *ANGPTL7* knockdown (Supplementary Fig. 20a, b) and overexpression (Supplementary Fig. 20c, d) in vitro. Through cell immunofluorescence staining, we found that overexpression of *ANGPTL7* exacerbated chondrocyte degeneration, whereas knockdown of *ANGPTL7* mitigated chondrocyte degeneration (Supplementary Fig. 20e–j). For example, knocking down the *ANGPTL7* gene expression in chondrocytes led to increased synthesis of ECM components, such as COL2A1 (Supplementary Fig. 20e) and ACAN (Supplementary Fig. 20f), and promoted the expression of cartilage differentiation gene *SOX9* (Supplementary Fig. 20g), while reducing the expression of ADAMTS5 (Supplementary Fig. 20h) and MMP13 (Supplementary Fig. 20i). Additionally, we co-cultured common chondrocytes and *ANGPTL7*-overexpressing chondrocytes with human umbilical vein endothelial cells (HUVECs), selectively adding FGF2 protein or Alofanib (a FGFR2 inhibitor). Through tube-forming experiments, we verified that *ANGPTL7*^+^ chondrocytes significantly enhanced the angiogenic ability of HUVECs. However, the angiogenic ability of *ANGPTL7*^+^ chondrocytes was weakened after the addition of Alofanib (Supplementary Fig. 20k, l).

### *Sparc*^+^ OBs negatively regulate bone mineralization and osteoblastic differentiation in KOA mice

**Fig. 9:**
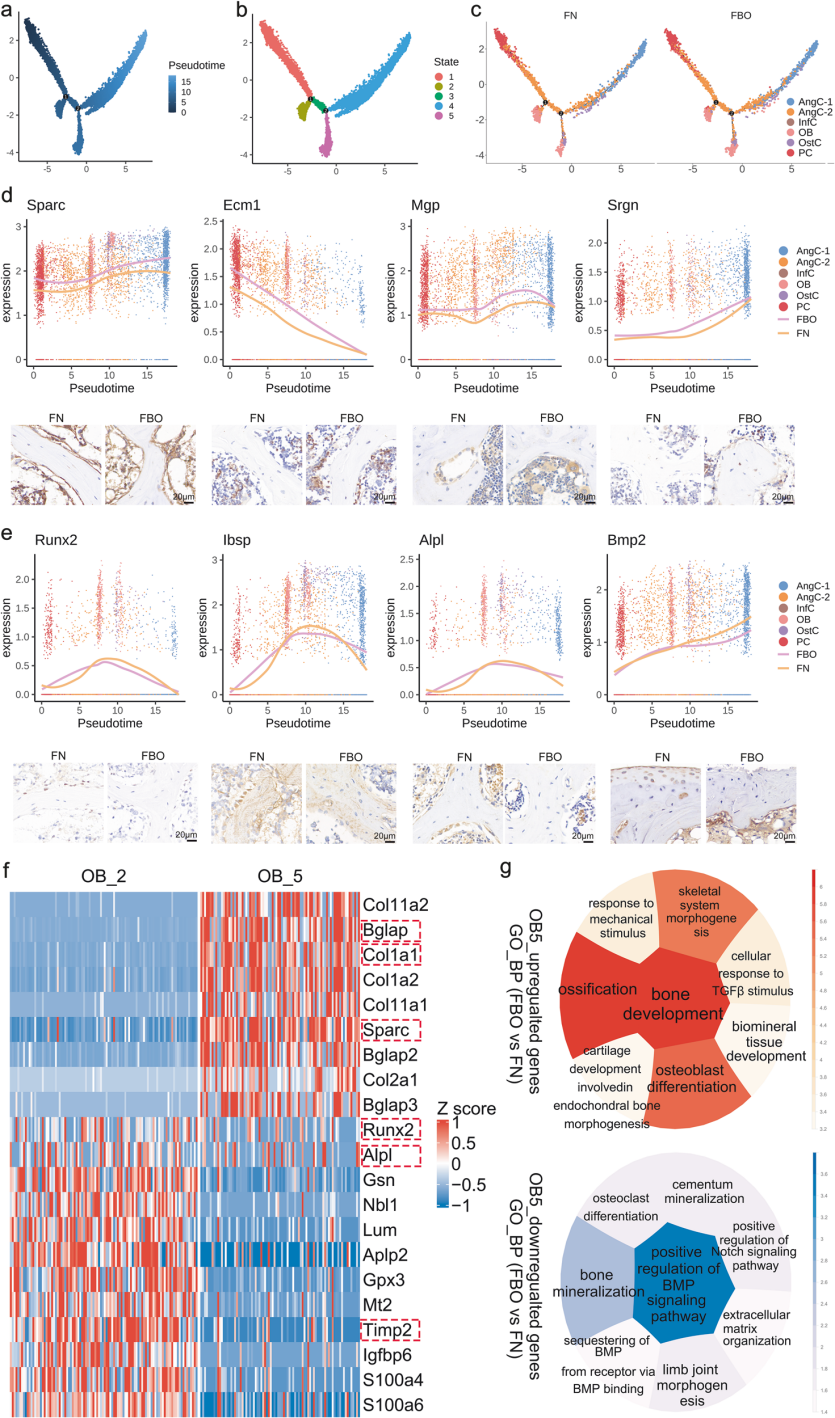
*Sparc*^+^ OBs negatively regulated bone mineralization and osteoblastic differentiation in KOA mice. **a**–**c** Developmental pseudotime for cells present along the trajectory inferred by Monocle 2. The trajectory plot shows the pseudotime, cell state, and cell subtypes of each cell, and choose PC cells as the start of time. **d** Increased *Sparc*, *Ecm1*, *Mgp*, and *Srgn* gene expression levels of all cells along cell trajectory in FBO sample compared with FN sample, which was highly related with the limited mineralization of trabecular bone. **e** Decreased *Runx2*, *Ibsp*, *Alpl*, and *Bmp2* gene expression levels of OB and OstC along cell trajectory in FBO sample compared with FN sample, which was highly related with the osteoblast development. All referred genes were confirmed in vivo by IHC staining, Scale bar = 20 μm. **f** Heatmap shows the differential expressed genes of state5 osteoblasts (OB5) and state2 osteoblasts (OB2) in FBO group. **g** The circle enrichment plot shows the GO database results of group FBO compared with group FN in OB5. The deeper the color is, or the bigger the size is, the −log_10_(*p*-value) is greater, indicating a more significant difference. Red (YlOrRd) circle plot indicates the up-regulation function of group FBO relative to group FN, and the blue (PuBu) circle plot indicates the down-regulation function

It was found that starting from PCs in FN and FBO samples, both can differentiate into different subtypes of OBs and CCs through trajectory analysis (Fig. 9a–c). While the expression levels of genes that negatively regulate bone mineralization such as *Sparc*, *Ecm1*, *Mgp*, and *Srgn* in each cell group during the differentiation process of PCs in FBO samples were always higher than those in FN samples, which were verified by IHC staining (Fig. 9d). It was suggested that there was a significant inhibition of subchondral bone mineralization in FBO mice. Furthermore, the expression levels of osteoblastic differentiation-related genes such as *Runx2*, *Ibsp*, *Alpl*, and *Bmp2* in some OBs and OstCs in FBO samples were significantly lower than those in FN samples, which were verified by IHC staining (Fig. 9e), suggesting that impaired osteogenic differentiation of subchondral bone in FBO mice.

OBs differentiated from PCs are naturally divided into two subgroups (Fig. 9c), and there are significant differences in gene expression of OBs in the two states. State 5 OBs (OB5) highly expressed genes such as *Sparc*, *Bglap*, and *Col1a1*, but lowly expressed genes such as *Runx2*, *Alpl*, and *Timp2* compared with state 2 OBs (OB2) (Fig. 9f). In addition, the number of OB5 increased, while the number of OB2 decreased in FBO sample compared with those in the FN sample (Supplementary Fig. 21). And GO enrichment analysis of the upregulated and downregulated genes of OB5 in the FBO sample compared to the FN sample, revealed that increased *Sparc*^+^ OB5 was highly related to the insufficient osteoblastic differentiation and subchondral bone mineralization in FBO mice (Fig. 9g). Therefore, it is suggested that *Sparc*^+^ OB5 potentially inhibited bone mineralization and osteoblastic differentiation during subchondral bone remodeling in FBO mice.

To further investigate the role of *Sparc*^+^ osteoblast in bone remodeling and KOA progression, we constructed osteoblast-like *MC3T3* cells models with *Sparc* knockdown (Supplementary Fig. 22a, b) and overexpression (Supplementary Fig. 22c, d) in vitro. Through cell immunofluorescence staining, we found that overexpression of *Sparc* mitigated osteoblast development, whereas knockdown of *Sparc* promoted osteoblast differentiation (Supplementary Fig. 22e–h). Specifically, knocking down the *Sparc* gene expression in osteoblast-like *MC3T3* cells led to increased expression of proteins coupling with osteoblast differentiation, including COL1A1 (Supplementary Fig. 22e) and OSTEOCALCIN (OCN, Supplementary Fig. 22f), and ALKALINE PHOSPHATASE (ALP, Supplementary Fig. 22g). Furthermore, we performed the Alizarin Red staining (ARS) for the osteoblast-like *MC3T3* cells treated with PBS, si-SPARC 3, and SPARC-overexpressing lentivirus. And it was found that osteoblasts with *Sparc* knocking down presented significantly bigger ARS positive area compared with the control and *Sparc*^+^ osteoblasts (Supplementary Fig. 22i, j).

### Positive correlation between the expression level of *ANGPTL7* and *SPARC* genes with KOA progression

Tibial plateau samples were further tested for the verification of the correlation between the expression of *ANGPTL7* and *SPARC* genes with KOA development. Specimens obtained from patients who had undergone above-knee amputation were classified as the normal group, while samples from patients with KOA who underwent total knee arthroplasty were categorized as the KOA group (Supplementary Fig. 23a). Immunohistochemical staining and semi-quantitative analysis revealed that the expression levels of ANGPTL7 in chondrocytes (Supplementary Fig. 23b, c) and SPARC in osteoblasts (Supplementary Fig. 23e, f) from KOA tibial plateau specimens were notably elevated compared to those in the normal control group. Furthermore, a correlation analysis was performed between the proportion of ANGPTL7 and SPARC-positive cells and their respective tissue OARSI scores. The results indicated a positive correlation between the gene expression levels and the tissue OARSI scores (Supplementary Fig. 23d,g). Specifically, higher expression levels of ANGPTL7 and SPARC genes correspond to a higher OARSI score, suggesting a more severe progression of KOA.

## Discussion

This study re-confirmed that pathological remodeling of subchondral bone plays a key role in KOA development.^25,26^ It was found that vascular invasion in the early stage of KOA led to a bottom-up degradation of the articular cartilage.^27^ Research by Xiao et al. reported that cartilage degradation of KOA might resemble an endochondral ossification process similar to that of the growth plate.^28^ Wang et al. found that pathological calcification of the cartilage presented a bidirectional pathological calcification process from the cartilage surface downward and from calcified cartilage upward.^29^ Therefore, the findings in this study suggest that cartilage degeneration in early KOA is a bidirectional process. For the late-stage KOA, the tidemark was destroyed with the cartilage tissue worn away. Additionally, granulation tissue and cartilage-like tissue infiltrated the subchondral trabeculae. Reines et al. found that the subchondral bone of inflammatory arthritis might undergo an autoregulatory repair process similar to limb development.^30^ Zhang et al. reported deposits of cartilaginous tissue within the trabeculae of exposed subchondral bone in KOA patients,^31^ which was further verified by immune-positively stained with COL2A1 and ACAN in this study. Therefore, it was induced that the deposited chondrocytes likely participated in subchondral bone remodeling in the form of endochondral ossification.

A postmenopausal KOA mouse model in bipedal mice has been successfully developed to understand the pathogenesis of KOA through single-cell transcriptomics. Previous study had shown that the bipedal mouse model was suitable for studying KOA.^32^ Son et al. demonstrated that obesity and forced bipedal walking on a specially designed treadmill can promote the development of KOA.^33^ During human evolution, the knee joint underwent adaptations in response to the biomechanical requirements of bipedalism through modifications in chondrocyte developmental programs, which may affect the risk of KOA.^34^ This study offers preliminary evidence, through FEA and in vitro cell experiments in limited conditions, that the exacerbation of KOA in bipedal mice is attributed to the increased mechanical stress on the knee joint. In addition, decreased subchondral bone mass due to estrogen signaling impairment worsens load-induced KOA development,^35^ and postmenopausal osteoporosis potentially aggravated KOA pathology by increasing subchondral osteoclast activity.^36^

Single-cell transcriptomics elucidates key changes in the progression of KOA. The previous findings including the classification of chondrocytes, such as effector chondrocytes, regulatory chondrocytes, and homeostatic chondrocytes,^37^ as well as senescent chondrocytes,^38^ pre-inflammatory and inflammatory chondrocyte population,^39^ iron-related chondrocyte subpopulations.^40^ Femoral condyle tissue, excluding the growth plate, was isolated for sc-RNA-seq and analysis in this study. A single-cell atlas of the osteochondral composite tissue, including CCs, ECs, OBs, PCs, and so on, was successfully constructed. In addition to the abovementioned InfCs, preHTCs, and AgeCs, this study discovered three novel subtypes of CCs, including OstCs and two different types of AngCs. Vascular invasion facilitates the formation of channels that connect the subchondral bone and cartilage, allowing for the migration of CCs. Some migrated CCs participated in subchondral bone remodeling and differentiated into cells that highly expressed angiogenic genes. Through cell interaction analysis and IF staining, this study further confirmed that *Angptl7*^+^ CCs (AngC-2) activate ECs via the FGF2 (ligand)-FGFR2 (receptor) interaction, mediating vessel growth and invasion.^41,42^

It has been reported that the growth of H-type vessels in the subchondral bone was closely related to KOA development.^43–45^ This study found that some of the proliferative vessels in the subchondral bone and cartilage were H-type vessels, and TEM revealed the recruitment of OBs around the vessel. This was possibly related to the increase in the number of osterix^+^ osteoprogenitor cells and sclerotic changes in the subchondral bone. Nevertheless, the trabecular bone exhibited signs of under-mineralization, resulting in compromised stress-bearing capacity and exacerbating the KOA development. Bianco et al. reported that reduced expression of bone sialoprotein in PCs in sclerotic areas of KOA subchondral bone leads to inadequate osteoblastic differentiation and mineralization of trabecular bone.^46^ While this study identified a subset of dysfunctional OBs in KOA subchondral bone, known as *Sparc*^+^ OBs. *Sparc*^+^ OBs exhibit decreased expression of osteogenic differentiation-related genes, including *Runx2*, *Ibsp*, and *Alpl*, and heightened expression of genes associated with inhibiting bone mineralization, such as *Sparc*, *Ecm1*, *Mgp*, and *Srgn*. It was suggested that *Sparc*^+^ osteoblast negatively regulated the bone mineralization and osteoblastic differentiation in KOA mice.

Additionally, this study had several limitations. First, the construction of the KOA mouse model requires a long period of time. The bipedal mouse model needs to be established at 3 weeks of age, followed by bilateral ovariectomy at 10 weeks of age, with significant KOA phenotypes only observed at 18 and 22 weeks of age. Second, the single-cell transcriptomics results of this study may only be applicable to certain subtypes of KOA characterized by subchondral bone remodeling and cannot yet be generalized to all KOA subtypes. Third, a longer observation period was still needed to detect and validate the late-stage characteristics and mechanisms of subchondral bone remodeling in the KOA mouse model. In conclusion, we constructed a postmenopausal KOA model in bipedal mice and generated a single-cell atlas of the osteochondral complex tissue. Three novel subpopulations of CCs, including *Smoc2*^+^ angiogenic CCs, *Angptl7*^+^ angiogenic CCs, and *Col1a1*^+^ osteogenic CCs were identified. *Angptl7*^+^ CCs may promote the growth and invasion of H-type vessels by interacting with ECs via Fgf2-Fgfr2 signaling pathway. The increase in H-type vessels recruited many Osterix^+^ PCs for promoting osteogenesis in subchondral bone remodeling. Nevertheless, *Sparc*^+^ OBs exhibited functional dysregulation throughout the process of osteoblastic differentiation and bone mineralization, leading to pathological remodeling of the subchondral bone. These discoveries offer novel avenues for potential therapeutic interventions in the treatment of KOA.

## Materials and methods

Detailed methods for all protocols used in this study are provided in the “Expanded materials and methods” in the Supplementary files.

### Acquisition of human tibial plateau samples

Five patients with primary KOA who had undergone knee replacement surgery were included in this study. The osteotomized tibial plateau specimen from each patient was obtained immediately after surgery and preserved in 4% paraformaldehyde at 4 °C for subsequent micro-CT measurement and histological evaluation. Informed consent has been obtained from all patients in the study, and the Biomedical Ethics Review Committee of West China Hospital, Sichuan University has granted approval for this study (No. 2022-243).

### Construction of KOA mouse model

The forelimbs and tail of 3-week-old C57 mice were cut off in a sterile environment, and exercises for the bipedal activities were performed on a treadmill with a slow speed. Then half of female normal and bipedal mice were bilateral ovariectomized via a midline incision for excising both ovaries at 10 weeks of age. KOA phenotype of experimental mice was evaluated once a month until 22 weeks of age. All animal experiments have been conducted to conform to the guidelines of the Experimental Animals Management Committee of Sichuan Province and approved by the Sichuan University Animal Care and Use Committee (No. 20230609002).

### Finite element analysis (FEA)

Three-dimensional model reconstruction of the knee joint was performed using Mimics software (Materialize, Belgium).^47^ Subsequently, the bones and articular cartilage were meshed using 4-node quadratic tetrahedron (C3D4) elements and 10-node quadratic tetrahedral (C3D10) elements respectively. The grid cell edge lengths of articular cartilage and bone were 10 μm and 35 μm, respectively. The meshed model was imported into Abaqus (Dassault Systemes, USA) for material assignment and FEA. Since this study was carried out on the basis of morphological changes rather than tissue material properties, articular cartilage was considered an isotropic linear elastic material (modulus of elasticity, *E* = 6 Mpa; Poisson’s ratio, *ν* = 0.49). Additionally, the femoral and tibial cortical bones are also considered to be isotropic elastic materials (*E* = 18,000 Mpa; *ν* = 0.3).^47,48^ The cancellous bone material was assigned in Mimics software using the nonlinear apparent density elastic modulus equation:$E={E}_{\max }* {(\frac{\rho }{{\rho }_{\max }})}^{2}{GPa}$ (*E*_*max*_ = 9.89 GPa, ${\rho }_{\max }=1.683g/{{cm}}^{3}$, *ν* = 0.3), the measurement of BMD of tibial was based on the Micro-CT gray value of each mouse.^49^ The articular cartilage is completely attached to the bone, and the contact between the femoral cartilage and the tibial cartilage is regarded as a frictionless hard contact with limited slippage and no penetration characteristics.^48^ In addition, during the simulation analysis, the femur was considered a rigid body controlled by the midpoint of the femoral transcondylar line. The joint bending angle of each group of models is guaranteed to be 150° to ensure the consistency of model analysis conditions. The tibia of mouse models was completely fixed, and a vertical load related to the weight of the mouse was applied to the femur (four-legged mice applied 1/4 of their body weight, and bipedal mice applied 1/2 of their body weight), to analyze and compare the strain and stress distribution of bone, and cartilage contact pressure in each model.

### Cell preparation for single-cell RNA sequencing and analysis

Ten femoral condyle tissue, excluding the growth plate, was isolated from the distal epiphyseal line of hindlimbs of 5 mice in each group at 22 weeks of age. Then the femoral condyles tissues were washed in ice-cold RPMI1640, and dissociated using Collagenase II (Sigma, V900892-100MG) and DNase I (Sigma 9003-98-9). Cell count and viability were estimated using fluorescence Cell Analyzer (Countstar^®^ Rigel S2) with AO/PI reagent after removal of erythrocytes (Solarbio R1010) and then debris and dead cells removal was decided to be performed or not (Miltenyi 130-109-398/130-090-101). Finally, fresh cells were washed twice in the RPMI1640 and then resuspended at 1 × 10^6^ cells per ml in 1×PBS and 0.04% bovine serum albumin. To increase the number of CD45-negative cells, the fresh cells were selected by flow cytometric sorting, and the CD45-positive and negative cells were mixed in a ratio of 1:1 before the examination.

### Statistical analysis

Statistical analysis was carried out by using the R package and GraphPad Prism software (V.9.3). The difference between groups was analyzed by Student’s *t*-test. Multiple group comparison was analyzed by One-way ANOVA followed by Tukey post hoc analysis. Pearson correlation coefficients were performed by correlation analysis. Of note, paired *t*-tests were performed for the quantitative analysis of subchondral bone parameters in Fig. 1, because each corresponding medial and lateral tibial plateau sample was from the same individual. Results were considered statistically significant when *p* < 0.05.

## Supplementary information

Supplementary material

Movie S1. Activities of bipedal mice in feeding cage

## Data Availability

All data associated with this study are present in the paper or the Supplementary Materials. All single-cell RNA sequencing data generated by this study are deposited in the Gene Expression Omnibus (GEO) database (http://www.ncbi.nlm.nih.gov/geo/). The data can be accessed under the accession number GSE267616.
