# Supplementary material for: Single-cell transcriptomics reveals novel chondrocyte and osteoblast subtypes and their role in knee osteoarthritis pathogenesis
Source: Signal Transduct Target Ther. 2025 Feb 5;10:40. doi: 10.1038/s41392-025-02136-8 (PMC11794573; doi:10.1038/s41392-025-02136-8)
Supplement: Supplementary file 1 — Supplementary material [file 41392_2025_2136_MOESM1_ESM.docx]

Supplementary Materials for

**Single-Cell Transcriptomics Reveals Novel Chondrocyte and Osteoblast Subtypes and Their Role in Knee Osteoarthritis Pathogenesis**

Insights from a Bipedal Postmenopausal KOA Mouse Model

Yuan Liu, Wacili Da, Ming-Jie Xu, Chao-Xin Xiao, Tao Deng, Sheng-Liang Zhou, Xiao-Ting Chen, Yao-Jia Zhou, Li Tang, Yong Nie, Yi Zeng, Hui-Qi Xie, Bin Shen

Correspondence to: [shenbin_1971@163.com](mailto:shenbin_1971@163.com), xiehuiqi@scu.edu.cn

**This PDF file includes:**

Materials and Methods

Figs. S1 to S23

Tables S1 to S6

Caption for Supplementary Movie. S1

**Other Supplementary Materials for this manuscript include the following:**

Movies S1

**Materials and Methods**

**Micro-CT measurement of human tibial plateau**

The microstructure of subchondral bone in tibial plateaus with KOA was assessed using a micro-CT imaging system (vivaCT 80, Scanco Medical AG, Switzerland). For quantitative analysis, the region of interest (ROI) was input into the SCANCO μCT Evaluation Program for analysis. Various parameters of bone microstructure were measured and compared, including trabecular bone volume fraction (BV/TV, %), bone mineral density (BMD, kg/m^2^), trabecular number (Tb. N, mm^-1^), trabecular thickness (Tb. Th, mm), trabecular separation (Tb. Sp, mm), and structure model index (SMI).

**Histological evaluation of human tibial plateau**

The sample taken from tibial plateau were fixed in 4% paraformaldehyde at 4℃ for 3 days, and followed by decalcification with 10% EDTA at pH 7.4 for approximately 1 month. The specimen was then embedded in paraffin, and coronally sliced with a thickness of 4 μm. The sections were then deparaffinized and hydrated before Hematoxylin and Eosin (H&E), Safranin O-Fast Green (SO&FG) and immunohistochemistry (IHC) staining. The vessels invaded into articular cartilage through the channel crossed tidemark was found in sections with H&E and SO&FG staining. CD31^hi^EMCN^hi^ type H vessels in tibial plateaus were determined through multiplex immunofluorescence staining. The cartilage-like tissue deposited in the subchondral bone, which was positively stained with safranin O.

**Histological evaluation of KOA mouse model**

The isolated knee specimens were fixed in 4% paraformaldehyde for 3 days and then decalcified in a 10% EDTA solution for 1 month at 37℃. After decalcification, the tissue specimens were embedded in paraffin, sectioned into 4-µm-thick sagittal sections. The sections were stained with H&E and SO&FG for histological analysis. The sections were scored by the Osteoarthritis Research Society International (OARSI) scoring criteria based on the results of the staining, and hyaline cartilage thickness was calculated to systematically quantify the healthy level of cartilage. Damage of cartilage ECM was determined by immunostaining for sections incubated with anti-Collagen II (abcam, ab34712) and anti-Aggrecan (ABclonal, A11691). Cells with positive staining was quantified using Image J software (J2).

**TEM observation**

For ultrastructural analysis, femoral condylar tissues of the mice were prefixed with a 3% glutaraldehyde, the tissue was then postfixed in 1% osmium tetroxide, dehydrated in series acetone, infiltrated in Epox 812 and embedded. The semi-thin sections were stained with methylene blue, and ultrathin sections were cut with diamond knife, stained with uranyl acetate and lead citrate. Finally, the sections were examined with a Transmission Electron Microscope (TEM, JEM-1400-FLASH, Japan).

**Gait analysis**

The gait analysis for the mice was conducted using an automated animal gait analysis system (VisuGait, Shanghai, China). The footprints from the spontaneous walking of mice were collected by placing each mouse individually in the walkway, allowing them to walk freely from one side to the other. Gait changes were recorded and analyzed using camera and VisuGait analysis software (Version 2.0). The recorded parameters including the walking cycle, stride length, walking speed, touchdown time and gait asymmetry index.

**Micro-CT measurement of mouse knees**

The knee joints of mice were scanned using a micro-computed tomography system (NMC-100, Ping sheng Medical Technology Co., LTD, China) with high resolution (30 mm) at 90 kV (0.06 mA). The obtained imaging data were subsequently reconstructed in 3D and analyzed for bone density using Recon 1.6.9.3 and Avatar 1.6.9.3. The data were statistically analyzed for subchondral bone-specific parameters, including BV/TV (%), BMD (g/cm^3^), Tb. Th (mm) and Tb. Sp (mm).

**InAlyzer examination**

The BMD and body composition (bone mineral content, fat mass, and lean mass) of mice were measured by InAlyzer equipment and dual X-Ray digital imaging software (Medikors Inc, Korea), based on the dual-energy x-ray absorptiometry (max: 80 kV, 1.25mA and 220VAC). Mice were anesthetized with isoflurane and placed in a supine position on testbed. And weight (g), BMD (g/cm^3^), fat and lean percent (%) of the whole body or knee joint (ROI selection) were measured and analyzed blindly.

**Mechanics experiment**

A mechanistic experiment was carried out using IonOptix C-Pace EM stretch system. Firstly, chondrocytes were plated in the PDMS chambers coated with fibronectin (50 μg/ml). The cells in the chamber were then stretched at 0%～10% strain with a stretching frequency of 0.2 Hz (4 ms). After 24 hours of stretching, the cells were stained with phalloidin to reveal the alterations of cytoskeleton. And RNA of stretched cells was extracted to determine the related gene changes. RT-PCR was employed to detect the mRNA expression of *PIEZO1*, *ADAMTS5*, *MMP13*, *COL2A1*, *ACAN*, and *SOX9*. The relative mRNA expression was calculated using the comparative cycle threshold (CT) method (ΔΔCT method), and all experiments were performed in triplicate. Detailed primer information for different genes was provided in Supplementary Table 3. Additionally, immunofluorescence staining was carried out for the aforementioned genes, and the mean optical density was measured by Image J (J2) software.

**Immunohistochemistry (IHC) staining**

The sections were dewaxed and treated with 1% hydrogen peroxide (Sigma-Aldrich) for 30 minutes. Antigen retrieval was achieved at 37 °C using 2 mg mL^−1^ hyaluronidase (Sigma-Aldrich). Following this, slides were blocked with 1.5% goat serum and incubated overnight at 4 °C with various primary antibodies. Detailed antibody information for various proteins was provided in Supplementary Table 4. Subsequently, they were exposed to secondary antibodies for 1 hour at room temperature, stained with 3,3′-diaminobenzidine (DAB, Vector Laboratories, Burlingame, CA, USA), and counterstained with hematoxylin.

**Multiplex IF staining**

Prepare paraffin sections, after dewaxing and rehydration, heat the EDTA repair solution to boiling and maintain above 95 ℃ for 30 minutes, naturally cool to repair antigens. IF staining for multiple antibodies was carried out sequentially using a multiplex immunofluorescence kit (IRISKit HyperView mIF kit, Minghong® LUMINIRIS®). Detailed antibody information for the proteins was provided in Supplementary Table 4. Olympus VS200 was used to capture images. And semi-quantitative analysis was performed through Qupath (Version 4.3) and Image J (J2) software.

**Single cell RNA-seq library construction and sequencing**

Single-cell RNA-Seq libraries were prepared by using a SeekOne^®^ Digital Droplet Single Cell 3’ library preparation kit (SeekGene Catalog No. K00202). Briefly, appropriate number of cells were mixed with reverse transcription reagent and added to the sample well on a SeekOne^®^ chip. Subsequently, Barcoded Hydrogel Beads (BHBs) and partitioning oil were separately dispensed into corresponding wells on the chip. After the generation of emulsion droplet, reverse transcription was performed at 42℃ for 90 minutes and inactivated at 80℃ for 15 minutes. Thereafter, cDNA was purified from broken droplet and amplified through PCR reaction. The amplified cDNA product was then cleaned, fragmented, end repaired, A-tailed and ligated to a sequencing adaptor. Finally, the indexed PCR were performed to amplified the DNA representing 3’ polyA part of expressing genes which also contained Cell Barcode and Unique Molecular Index. The indexed sequencing libraries were cleanup with SPRI beads, quantified by quantitative PCR (KAPA Biosystems KK4824) and then sequenced on illumina NovaSeq 6000 with PE150 read length.

**Data preprocessing**

Count data were imported into the Seurat single‐cell analysis software (v4.1.3; https://github.com/satijalab/seurat), and quality control of the sequenced libraries was carried out by removing the outlier cells and genes. Seurat was used to perform the downstream analysis following the standard pipeline using cells with more than 250 genes, and expressing < 10% mitochondrial genes were retained, resulting in 28158 genes × 65491 cell matrix for further analysis.

**Analysis and visualization of the processed sequencing data**

We have used the functions in the Seurat package to normalize and scale the single-cell gene expression data. It was first normalized by “NormalizeData” function with setting normalization method as “LogNormalize”. In detail, the expression of each gene *i* in cell *j* was determined by the UMI count of gene *i* divided by the total number of UMI of the cell *j*, followed by multiplying 10000 for the normalization and the log-transformed counts were then computed with base as 2. We then restricted the corrected expression matrix to the subsets of highly variable genes (HVGs), and centered and scaled the values before performing dimension reduction and clustering on them. Methodologically, the highly variable genes (HVGs) were selected based on vst method in function “FindVariableGenes”, and top 2000 HVGs were left for downstream analysis. All samples were integrated by the R package harmony. We then used the “RunPCA” function in the Seurat package to perform the principle component analysis (PCA) on the single-cell expression matrix with genes restricted to HVGs. Given that many principle components may lead to the very low proportion of the variance, the signal-to-noise ratio can be improved substantially by selecting a subset of significant principle components. The number of significant principal components was determined with a permutation test, implemented by permutationPA function from the jackstraw R package. The analysis had identified 25 significant principal components and only scores from these principle components were used for further analysis. We then utilized the “Find Clusters” function in the Seurat package to conduct the cell clustering analysis through embedding the cells into a graph structure in a PCA space.

Clustering at a resolution of 0.8 was performed on PCA‐reduced expression data for the top 25 principal components using the graph‐based shared nearest neighbor method (SNN), which calculated the neighborhood overlap (Jaccard index) between every cell and its nearest neighbors. Clustering results were visualized using uniform manifold approximation and projection (umap). Individual samples and sample groups were also visualized using umap. The marker genes for every cluster compared with the remaining cells were identified using the FindAllMarkers function. For each cluster, genes were selected such that they were expressed in at least 25% of cells with at least 0.25-fold difference.

**Cell Clusters Annotation and Data Visualization**

Cell type annotation was performed based on the collected bone marker genes, which leverages reference transcriptomic datasets of known cell types, was used to annotate the identities of various cell clusters. The umap was applied to visualize the single cell transcriptional profile in a 2D space. Other bar plots, boxplots, violin plots and heatmaps were generated by customized R code through ggplot2 (*v*3.4.3) and ComplexHeatmap (*v*2.14.0).

**Functional Enrichment Analysis**

To assess the function of identified cell marker genes, an enrichment test was conducted using R package cluster profiler (*v*4.4.4) with default settings. Gene ontology terms or KEGG pathways having adjusted *p*-value of less than 0.05 calculated by the hypergeometric test followed by the Benjamini-Hochberg method were defined as significant enriched term/pathways. Top 20 enriched term/pathways (ordered by ascending unadjusted *p* value) were chosen to be visualized.

To score individual cells for pathway activities, we used the R package AUCell. For each cell, an expression matrix was used to compute its gene expression rankings with the AUCell_build Rankings function with default parameters. The canonical pathway database was downloaded from the GO and KEGG website, and canonical pathway gene sets were used to score each cell. For each gene set and cell, area-under-the-curve (AUC) values were computed (AUCell_calcAUC function) based on gene expression rankings, where AUC values represented the fraction of genes within the top-ranking genes for each cell that are defined as part of the pathway gene set.

**Identification of sub-populations of chondrocytes**

Chondrocytes were divided into 6 clusters under resolution 0.2 by dimensionality reduction clustering. Subtyping of the chondrocytes are based on the highly expressed genes of each cluster. First, we used “FindAllmarker” to find top genes of each cluster, and then used GO/KEGG/Wikipath databases to found the specific biological functions of each cluster and named them.

**Cell-cell interaction analysis**

Cell-cell interaction from scRNA-seq data was predicted using CellChat. To validate the ligand-receptor interactions between Chondrocyte, Reticular cells, Progenitor cells, Osteoblast and Endothelial cells, we applied CellChat to further identify cellular communications. Normalized count matrix along with cell annotation metadata was processed through the standard CellChat pipeline, except that the communication probability was calculated with a truncated mean of 10%.

**Trajectory analysis**

Single-cell pseudotime trajectories were reconstructed using the R package monocle2. Dimensionality reduction was first performed with the DDRTree algorithm, using the expression of all highly variable genes detected as described above in “scRNA-seq analysis”. Cell trajectory was then captured using the orderCells function, with the starting pseudotime state denoted as the end of the trajectory that was found to be enriched for progenitor cells clusters.

**Cell Transfection**

*ANGPTL7* and *Sparc* was transfected into the human chondrocytes and osteoblast-like *MC3T3* cells (at 70% confluence) using lentivirus from GenePharma. The lentivirus was transfected by following the manufacturer’s instructions, with the efficiency monitored by qPCR and western blotting analyse. The human chondrocytes and osteoblast-like *MC3T3* cells (at 70% confluence) were transfected with 50 nM small interfering RNA (siR)–negative or siRNA-*ANGPTL7*, siRNA-*Sparc* (Tsingke Biotechnology) using Hieff Trans in vitro siRNA Transfection Reagent (YEASEN) by following the manufacturer’s protocols. The cells were harvested 48 hours later to confirm the efficiency of knockdown by qPCR and western blotting analyse.

**Western Blotting**

Proteins from Chondrocytes or osteoblast-like *MC3T3* cells under different treatments were extracted using Radioimmunoprecipitation assay buffer with 1 mM phenylmethanesulfonylfluoride (Beyotime Biotechnology), and the total protein concentration was determined using a BCA kit (Pierce). The extracted proteins were then mixed with a loading buffer (Pierce) and boiled at 100 ℃ for 8 minutes. Equal amounts of protein were separated by 12.5% sodium dodecyl sulfate polyacrylamide gel electrophoresis and transferred to a polyvinylidene difluoride membrane (Hybond). After blocking in 5% skim milk for 1 hour at room temperature, the membrane was probed with primary antibodies (Supplementary Table 4) at 4℃ overnight. The membrane was then incubated with secondary antibodies (ABclonal; 1:5000) at 37℃ for 1 hour. Chemiluminescent signals were generated using an enhanced chemiluminescence (ECL) imaging kit (Thermo Fisher Scientific). GAPDH was used as the internal control. The intensity of the bands was quantified using Image Processing and Analysis in Java (ImageJ; National Institutes of Health) software.

**Immunofluorescence Staining**

Chondrocytes or osteoblast-like *MC3T3* cells were seeded in a 24-well plate and incubated in a cell culture incubator for 24 h. Afterward, the cells were divided into the following groups: control group, gene knockdown group, and over-expression group. After the incubation period, the cells were fixed with 4% paraformaldehyde for 15 minutes. Subsequently, they were treated with a blocking solution containing 0.1% Triton X-100 for 15 min. The cells were then incubated overnight at 4 ℃ with primary antibodies (Supplementary Table 4). After washing the cells three times with PBS, they were incubated with appropriate secondary antibodies (1:400) for 1 h. Finally, the cell nuclei were stained with DAPI for 5 min at room temperature. The cells were observed using a fluorescence microscope.

**Human Umbilical Vein Endothelial Cell Tube Formation Assay**

After co-culture with human chondrocytes under different treatment, human umbilical vein endothelial cells (HUVECs) (7×10^5^) were seeded into 24-well plates coated with Matrigel 356234 (20 μL per well; BD Biosciences). After 8 hours of incubation at 37℃, the tube formation was visualized under a microscope (Nikon Corporation). The number of junctions of the associated tubes was calculated and compared among various groups.

**Alizarin red S (ARS) staining**

Alizarin red S (ARS) staining was carried out after 21 days of differentiation, after which the osteoblast-like *MC3T3* cells were washed with PBS, fixed with 4% paraformaldehyde for 10 min, and then incubated with 2% ARS solution (Solarbio, Beijing, China). After washing with PBS, the stained cells were examined using an inverted microscope. The fractions ARS^+^ areas were calculated using ImageJ software.


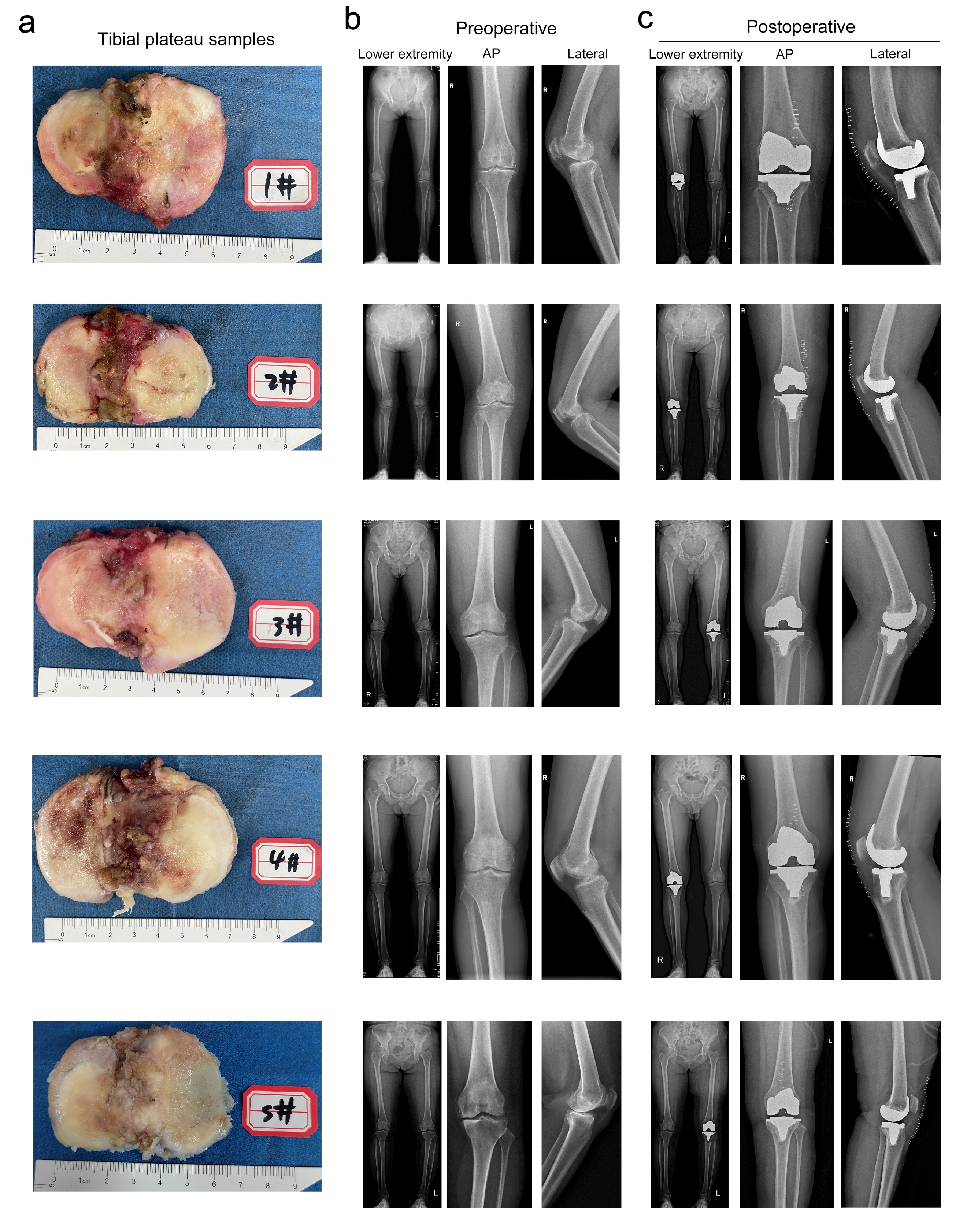


**Figure. S1.** Macroscopic images of tibial plateaus and radiographs of knees of representative KOA patients. **a** Macroscopic image of tibial plateaus from representative patients included in this study. **b** Full-length standing anteroposterior (AP) radiographs of lower extremities, AP and lateral radiographs of affected knees before TKA surgery. **c** Full-length standing AP radiographs of lower extremities, AP and lateral radiographs of the affected knees after the TKA surgery.


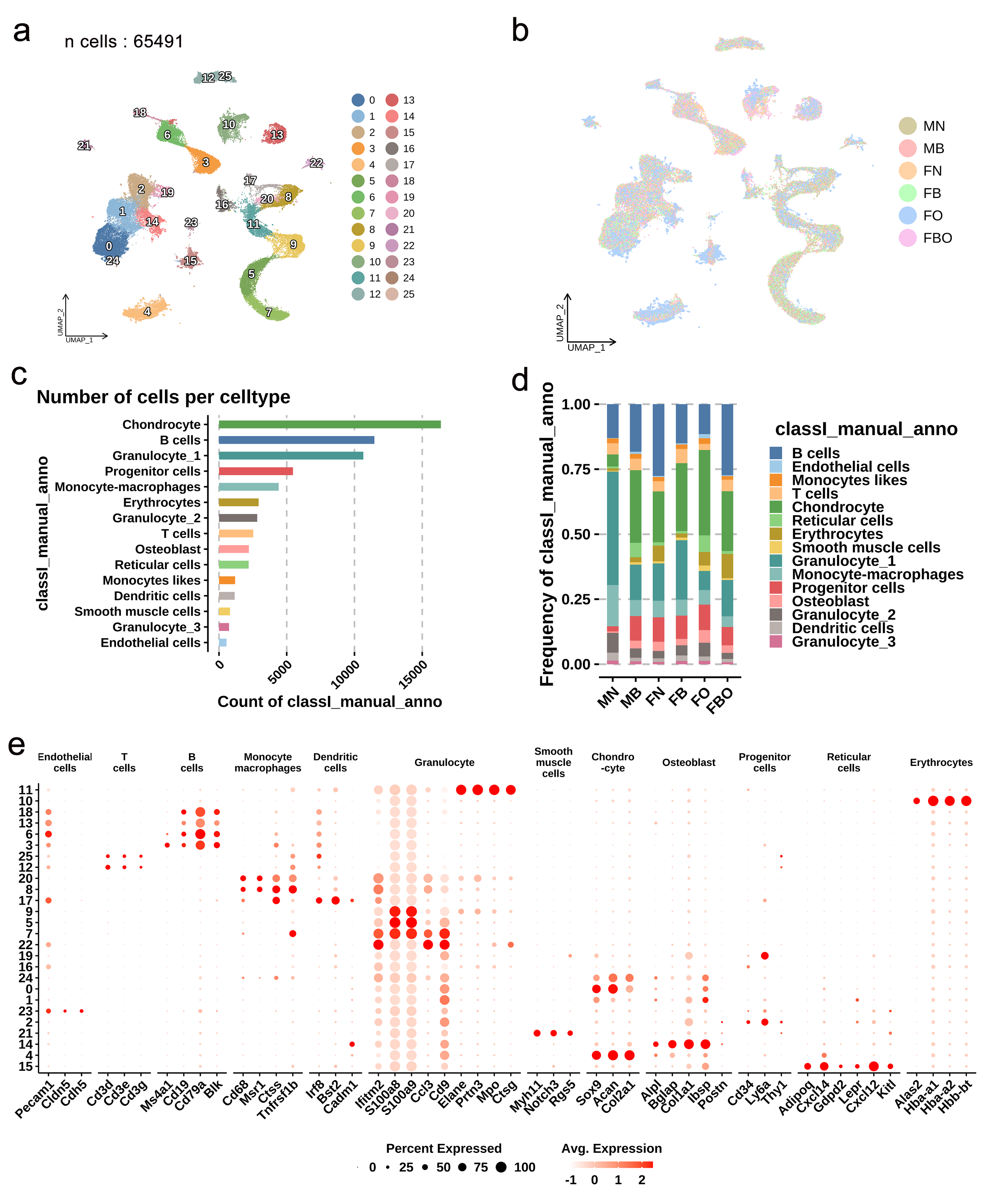


**Figure. S2.** ScRNA-seq atlas of cell clusters of femoral condyles for the six groups at 22 weeks of age. **a** UMAP plot of single cells profiled in the presenting work colored by clusters. **b** Uniform manifold approximation and projection (UMAP) plot showing the clusters distribution in the merged data set, which was colored by sample group. **c** Numbers of various cell types of the knee joints from the six groups. **d** Proportion of various cell types from the knee joints among the six groups. **e** Dot plot showing the expression of specific signatures in identified cell types. The dot color and size represent the mean expression and proportion of each cell population expressing genes, respectively.

**
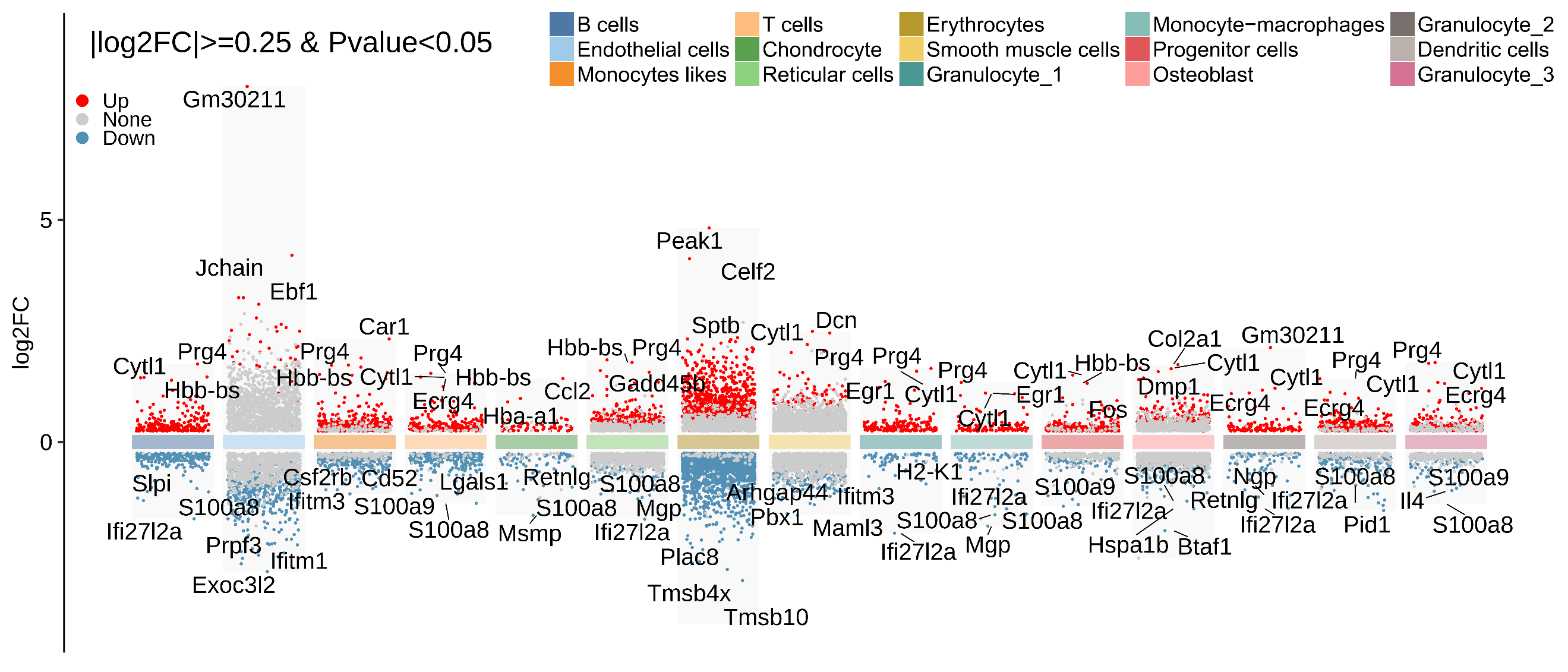
**

**Figure. S3.** The volcano plot of multi-group differential analysis shows the results of differential gene analysis in 15 cell groups between the MB and the MN group. Each color block on the horizontal axis represents a cell type, and the vertical axis represents the difference fold (log_2_Fold Change). The farther the point is from the X-axis, the greater the difference fold. The color of the dots indicates significant differences. We consider that genes with the average log_2_FC more than 0.25, and a *p* value less than 0.05 are significantly differentially expressed. Red dots indicate significantly up-regulated genes, blue dots indicate significantly down regulated genes, and gray dots indicate no significant differences.


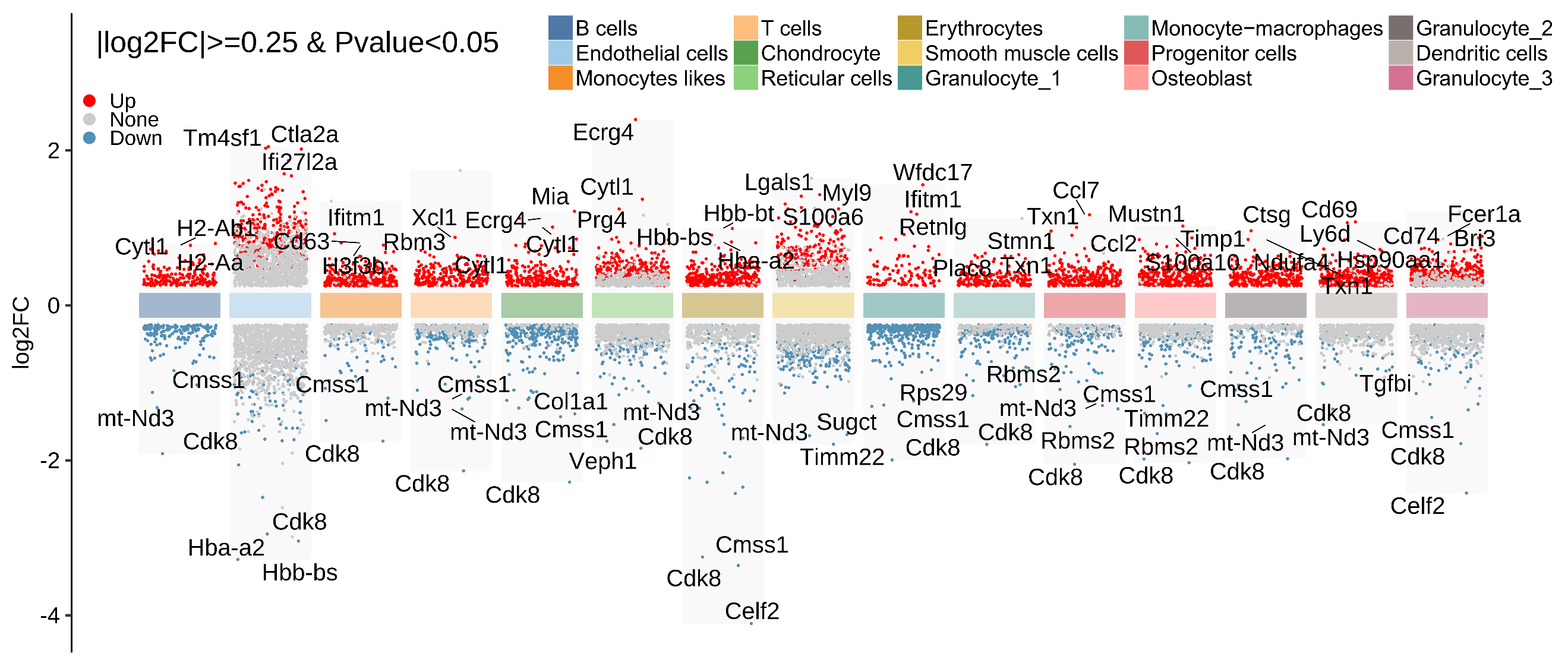


**Figure. S4.** The volcano plot of multi-group differential analysis shows the results of differential gene analysis in 15 cell groups between the FB and the FN group.


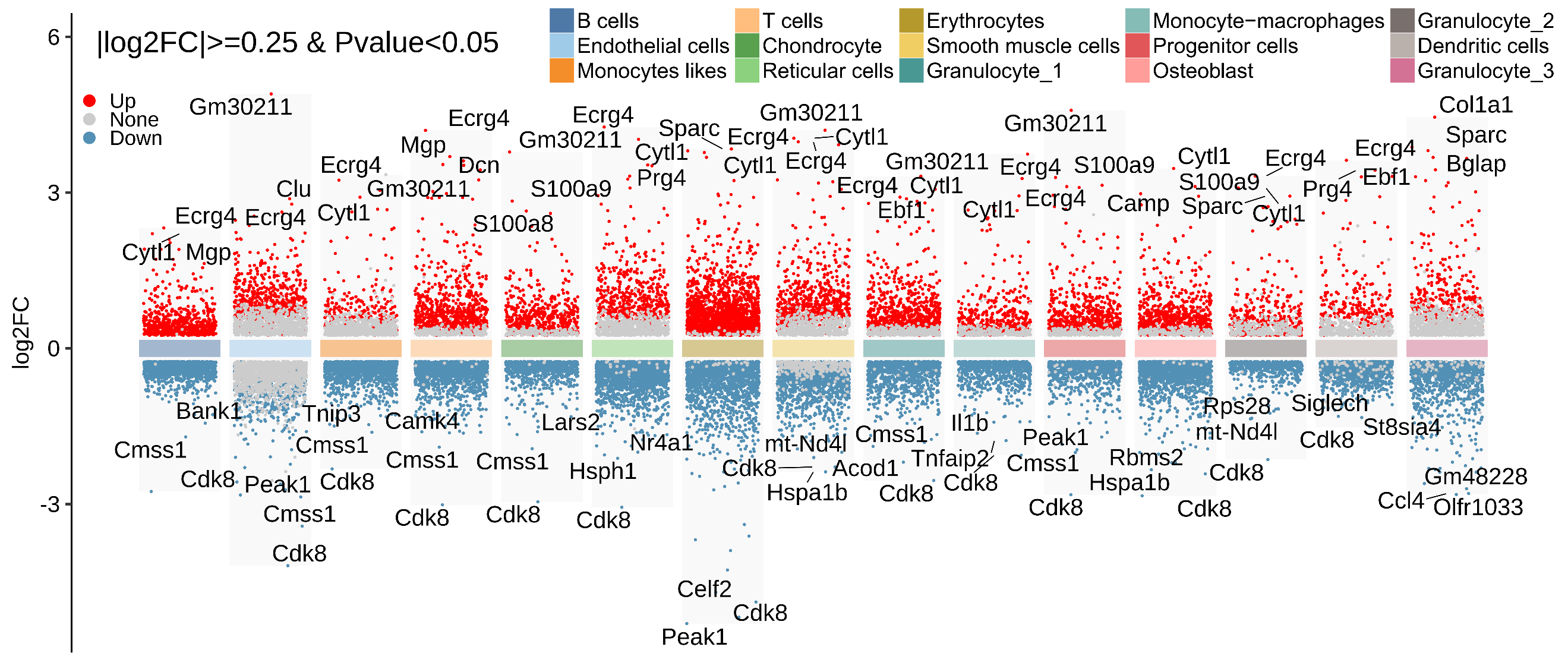


**Figure. S5.** The volcano plot of multi-group differential analysis shows the results of differential gene analysis in 15 cell groups between the FO and the FN group.


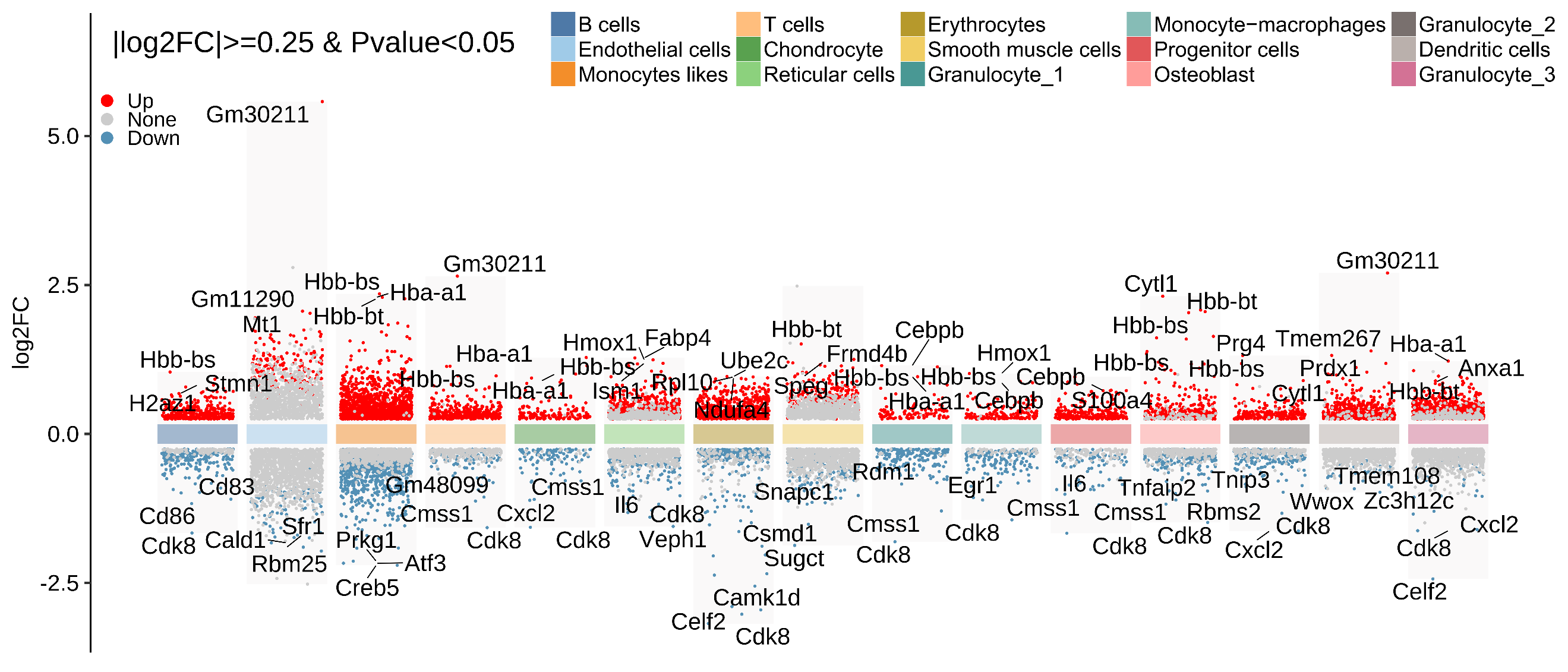


**Figure. S6.** The volcano plot of multi-group differential analysis shows the results of differential gene analysis in 15 cell groups between the FBO and the FN group.


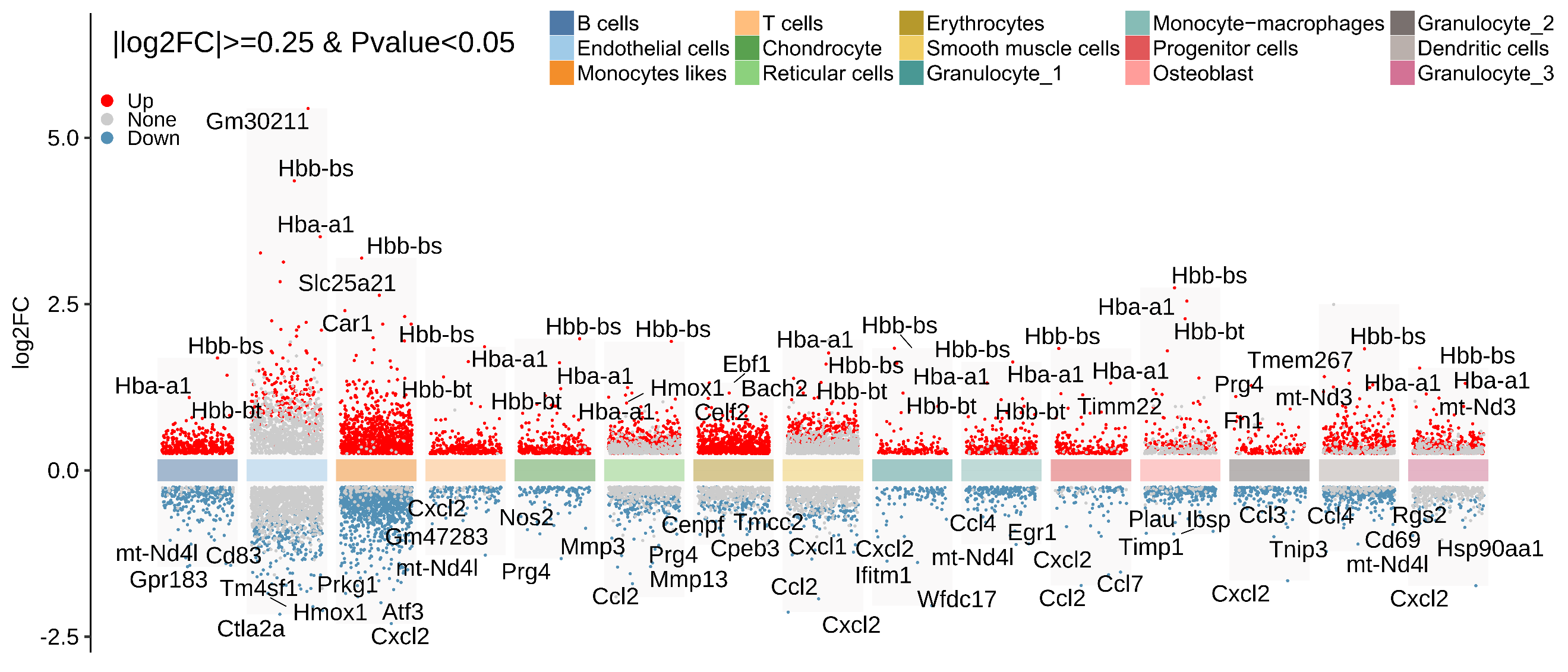


**Figure. S7.** The volcano plot of multi-group differential analysis shows the results of differential gene analysis in 15 cell groups between the FBO and the FB group.


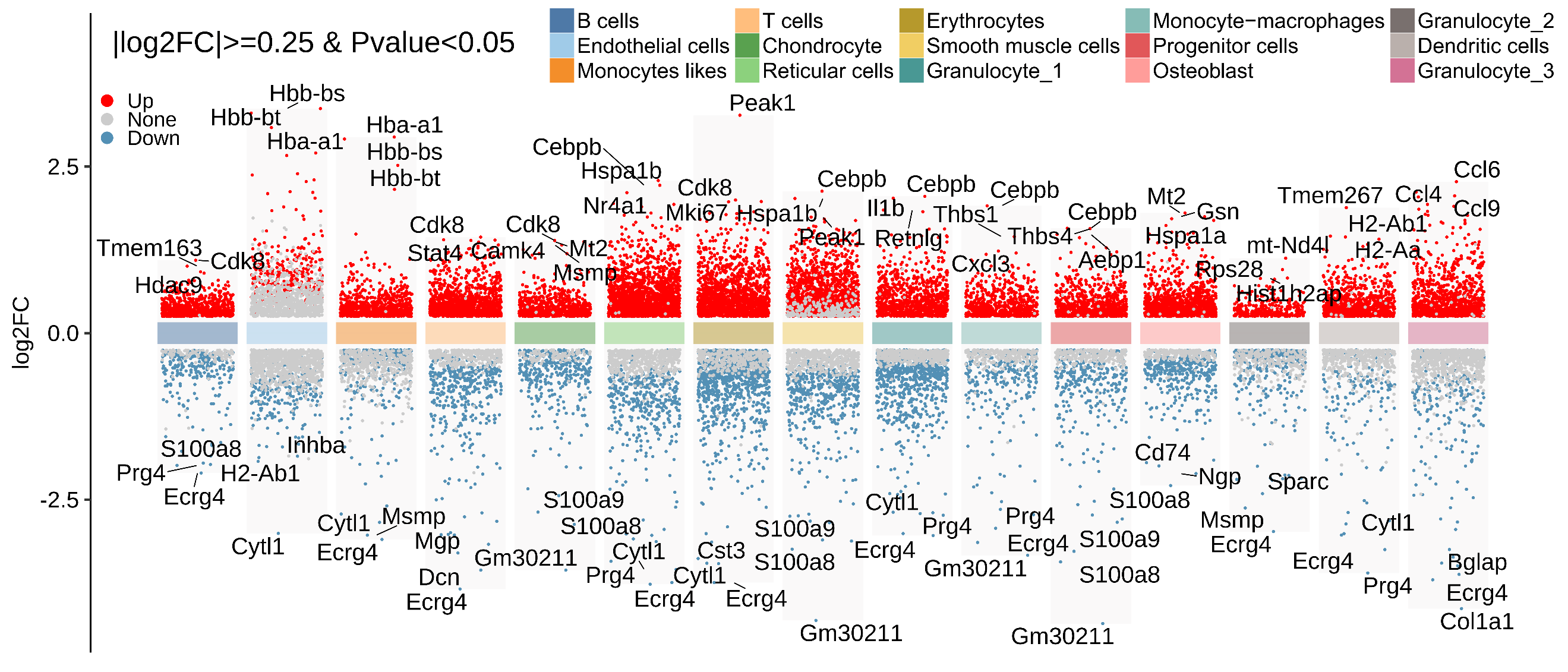


**Figure. S8.** The volcano plot of multi-group differential analysis shows the results of differential gene analysis in 15 cell groups between the FBO and the FO group.


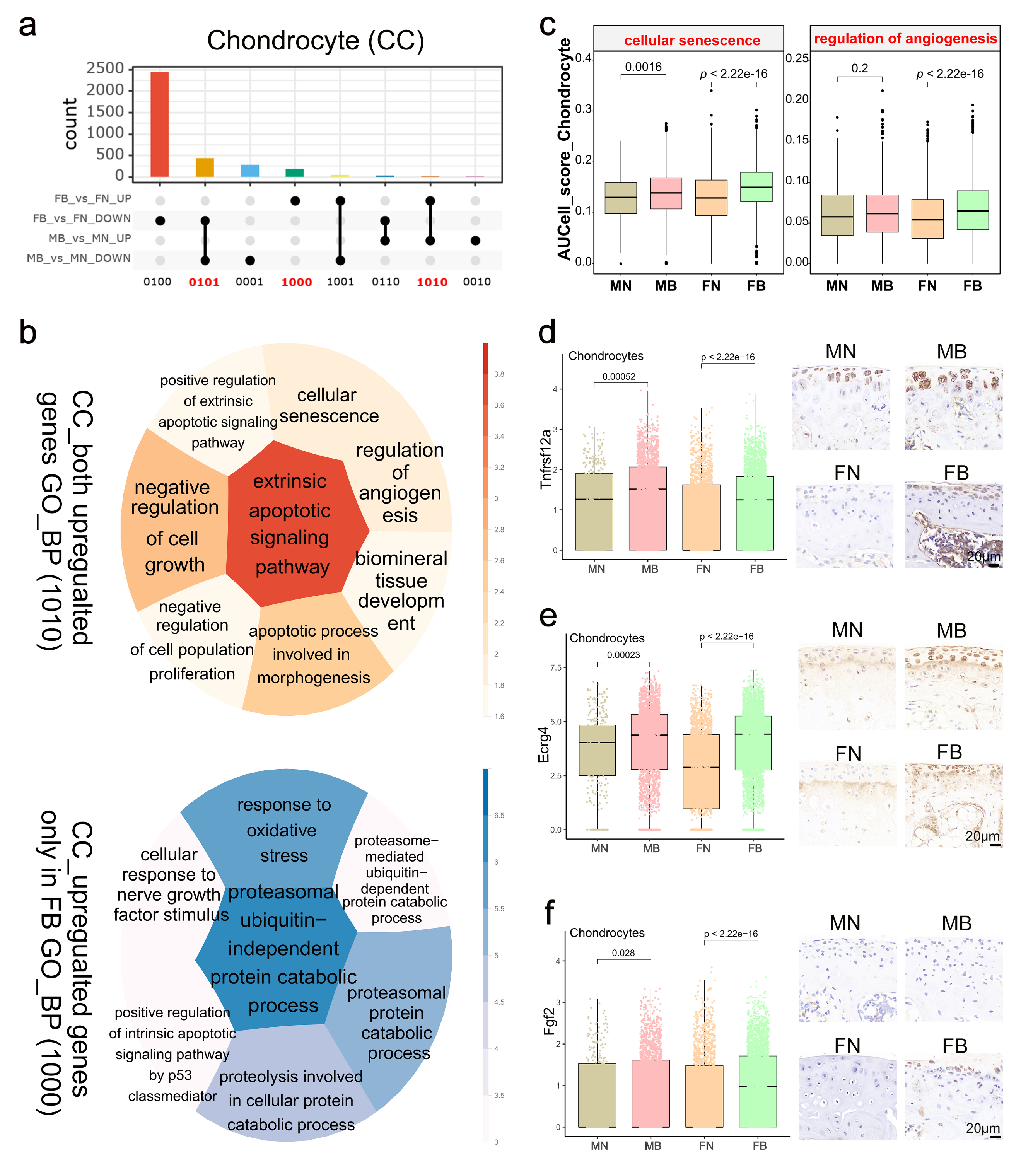


**Figure. S9.** Comparison of chondrocytes in female bipedal mice compared with the male group. **a** The VENN analysis results of upset were analyzed by the intersection set of gender differences among chondrocyte. Number 0 (No) and 1 (Yes) were used to indicate genes upregulated or downregulated in FB or MB group compared with FN or MN group. For example, 1010 refers to genes both upregulated in FB and MB group compared with FN and MN group, 1000 indicates genes only upregulated in FB vs FN comparison. **b** The circle enrichment plot shows the GO database pathways of genes regulated similarly or differently by the intersection set of gender differences among CC. The deeper the color is, or the bigger the size is, the -log_10_(*p*-value) is greater, indicating a more significant difference. **c** The box plot represents the scores of chondrocytes in normal and bipedal mice for “cellular senescence” and “regulation of angiogenesis” pathway in the GO database, scores come from the R package `AUcell`. **d-e** The box plot represents the *Tnfrsf12a* gene (**d**) *Ecrg4* gene (**e**) expression of chondrocytes related to the “cellular senescence” pathway in figure c. **f** The box plot represents the *Fgf2* gene expression of chondrocytes related to the “regulation of angiogenesis” pathway in figure c. All referred genes were confirmed *in vivo* by IHC staining, Scale bar = 20 μm.


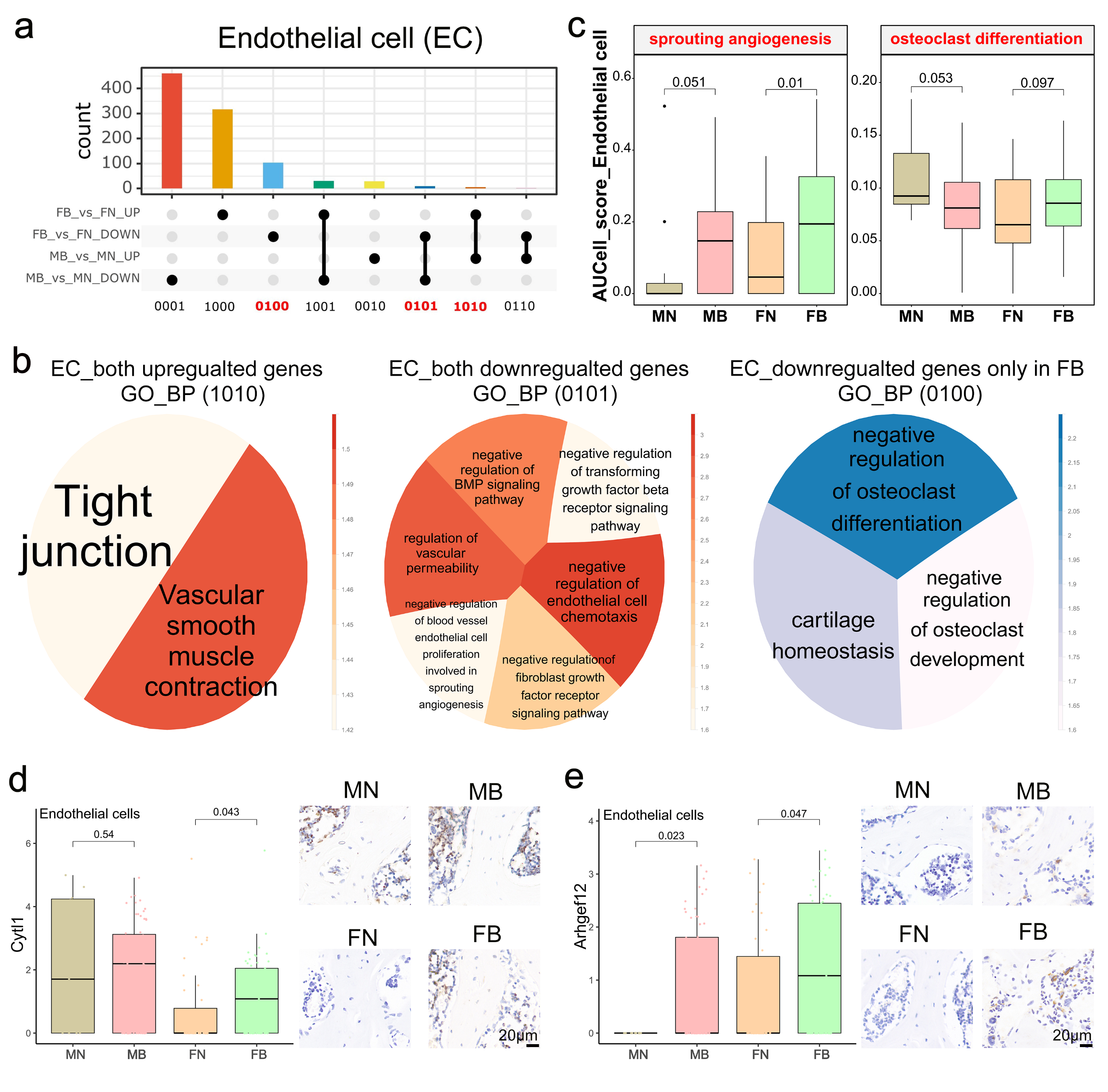


**Figure. S10.** Comparison of endothelial cells in female bipedal mice compared with the male group. **a** The VENN analysis results of upset were analyzed by the intersection set of gender differences among endothelial cell. Number 0 (No) and 1 (Yes) were used to indicate genes upregulated or downregulated in FB or MB group compared with FN or MN group. For example, 1010 refers to genes both upregulated in FB and MB group compared with FN and MN group, 1000 indicates genes only upregulated in FB vs FN comparison. **b** The circle enrichment plot shows the GO database pathways of genes regulated similarly or differently by the intersection set of gender differences among EC. The deeper the color is, or the bigger the size is, the -log_10_(*p*-value) is greater, indicating a more significant difference. **c** The box plot represents the scores of endothelial cells in normal and bipedal mice for “sprouting angiogenesis” and “osteoclast differentiation” pathway in the GO database, scores come from the R package `AUcell`. **d-e** The box plot represents the *Cytl1* gene (**d**) *Arhgef12* gene (**e**) expression of endothelial cells related to the “sprouting angiogenesis” pathway in figure c. All referred genes were confirmed *in vivo* by IHC staining, Scale bar = 20 μm.


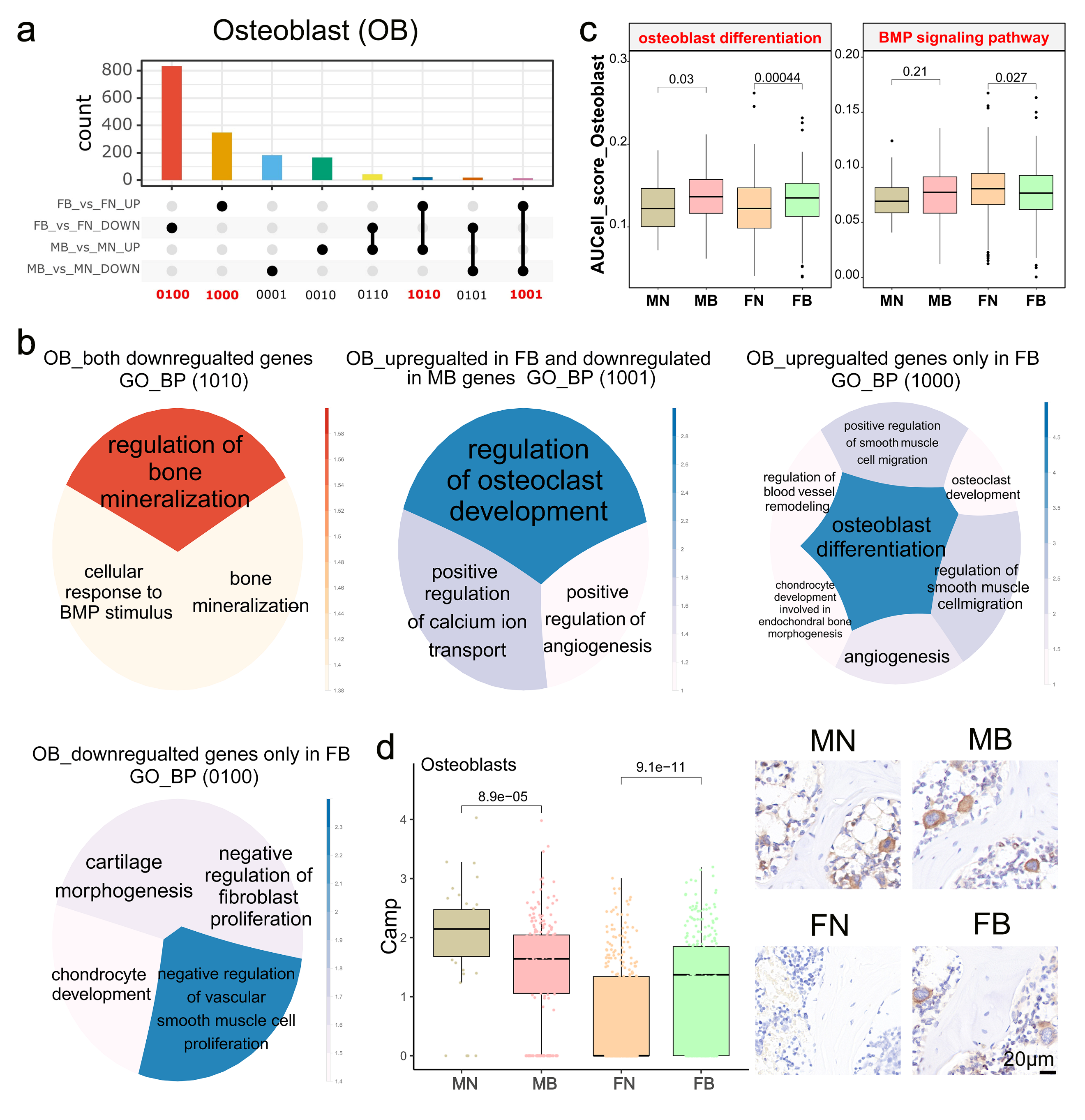


**Figure. S11.** Comparison of osteoblasts in female bipedal mice compared with the male group. **a** The VENN analysis results of upset were analyzed by the intersection set of gender differences among osteoblast**s**. Number 0 (No) and 1 (Yes) were used to indicate genes upregulated or downregulated in FB or MB group compared with FN or MN group. For example, 1010 refers to genes both upregulated in FB and MB group compared with FN and MN group, 1000 indicates genes only upregulated in FB vs FN comparison. **b** The circle enrichment plot shows the GO database pathways of genes regulated similarly or differently by the intersection set of gender differences among OB. The deeper the color is, or the bigger the size is, the -log_10_(*p*-value) is greater, indicating a more significant difference. **c** The box plot represents the scores of osteoblasts in normal and bipedal mice for “osteoblast differentiation” and “BMP signaling pathway” pathway in the GO database, scores come from the R tool `AUcell`. **d** The box plot represented the *Camp* gene expression of osteoblasts related to the “osteoblast differentiation” pathway in figure c. All referred genes were confirmed *in vivo* by IHC staining, Scale bar = 20 μm.


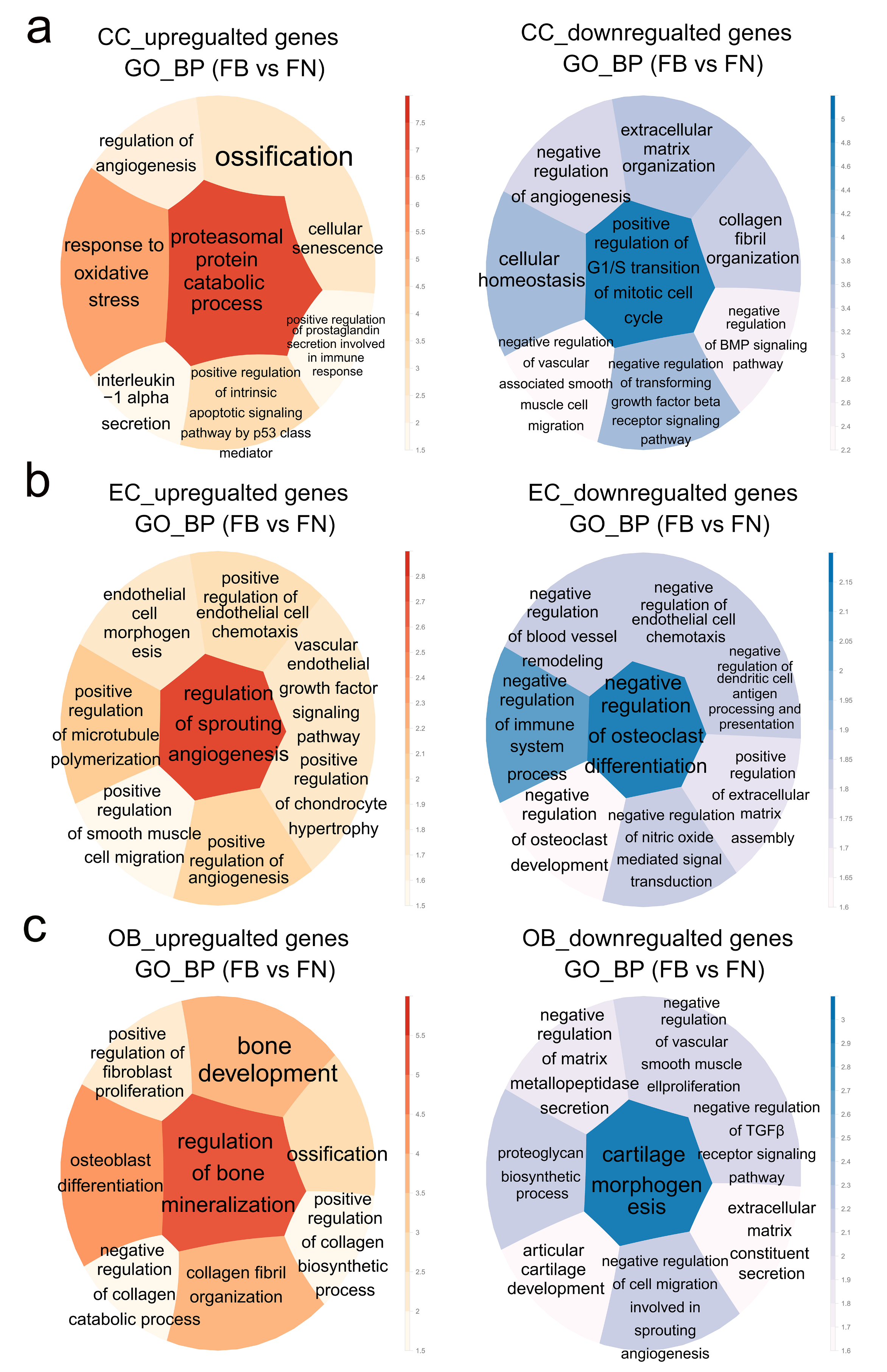


**Figure. S12.** The circle enrichment plot shows the GO database results for the group FB compared with the group FN in chondrocytes **(a)**, endothelial cells **(b)** and osteoblasts **(c)** The deeper the color is, or the bigger the size is, the -log_10_(*p*-value) is greater, indicating a more significant difference. Red (YlOrRd) circle plot indicates the up-regulation function of group FB relative to group FN (left), and the blue (PuBu) circle plot indicates the down-regulation function (right).


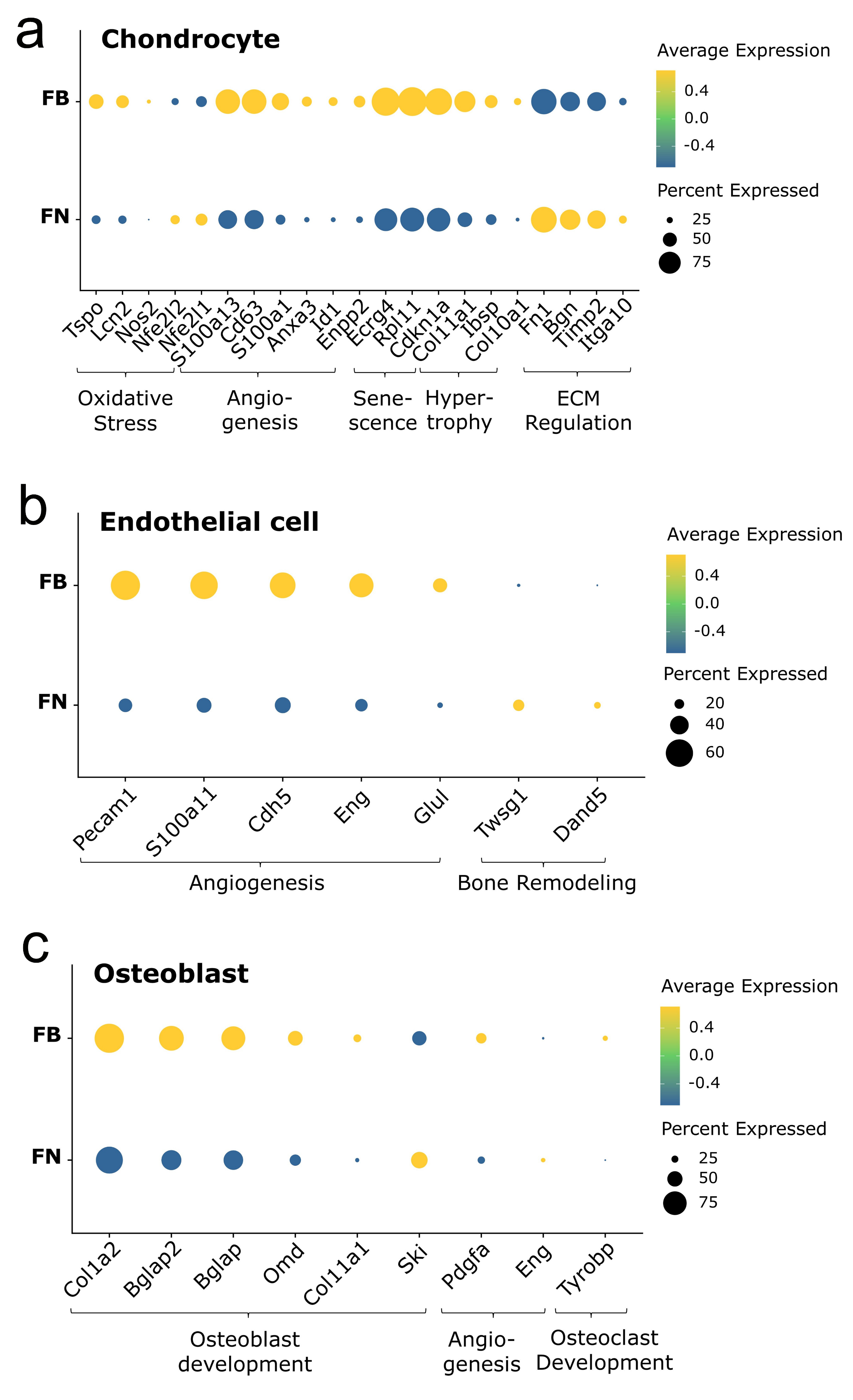


**Figure. S13.** Dot plots indicate differentially expressed genes in the chondrocytes **(a)**, endothelial cells **(b)** and osteoblasts **(c)** between the FB and FN groups. Presented genes are derived from the results of interest through the enrichment analysis, mostly referring to degeneration of chondrocytes, angiogenesis and remodeling of subchondral bone.


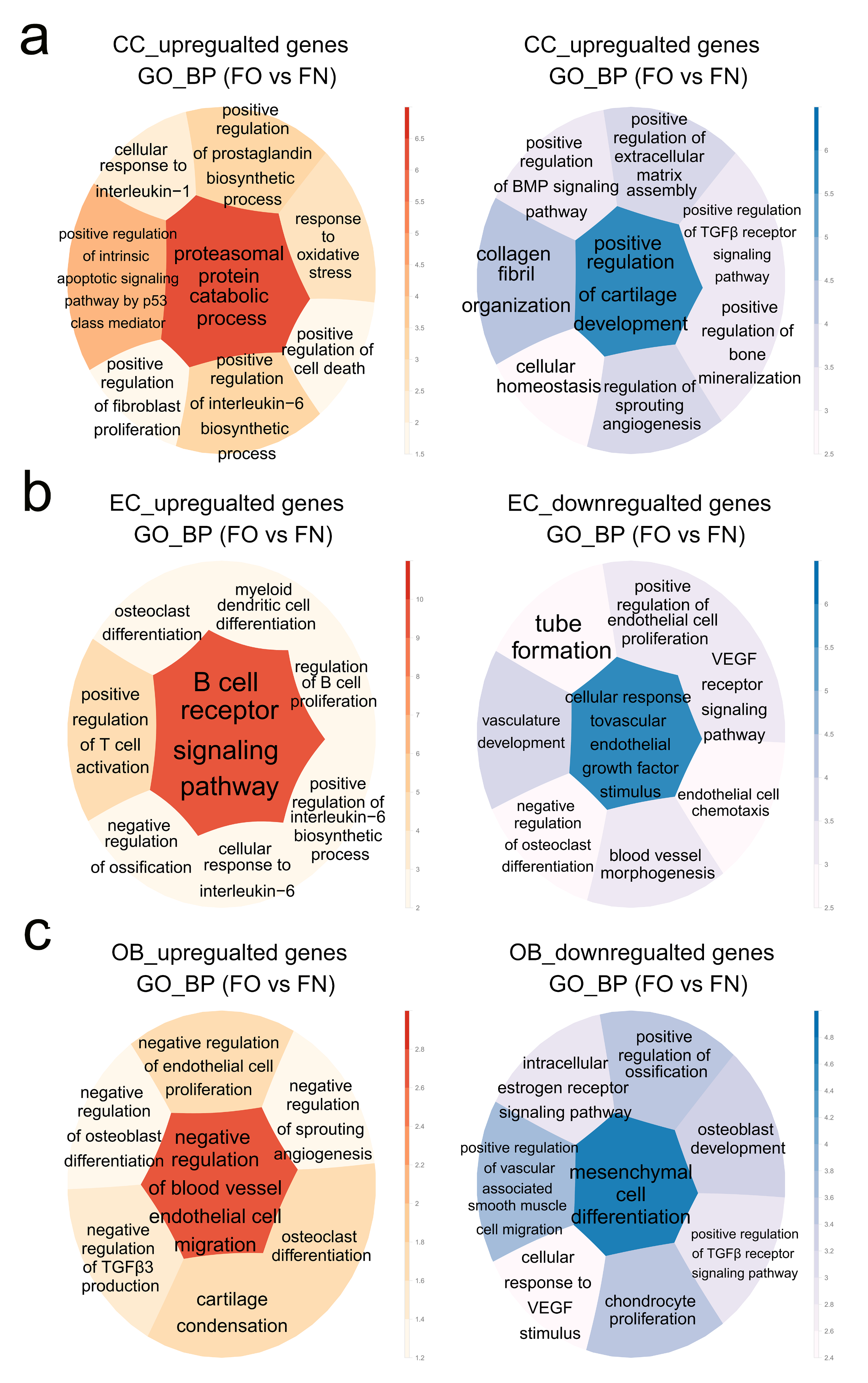


**Figure. S14.** The circle enrichment plot showed the GO database results of group FO compared with group FN in chondrocytes **(a)**, endothelial cells **(b)** and osteoblasts **(c)** The deeper the color is, or the bigger the size is, the -log_10_(*p*-value) is greater, indicating a more significant difference. Red (YlOrRd) circle plot indicates the up-regulation function of group FO relative to group FN (left), and the blue (PuBu) circle plot indicates the down-regulation function (right).


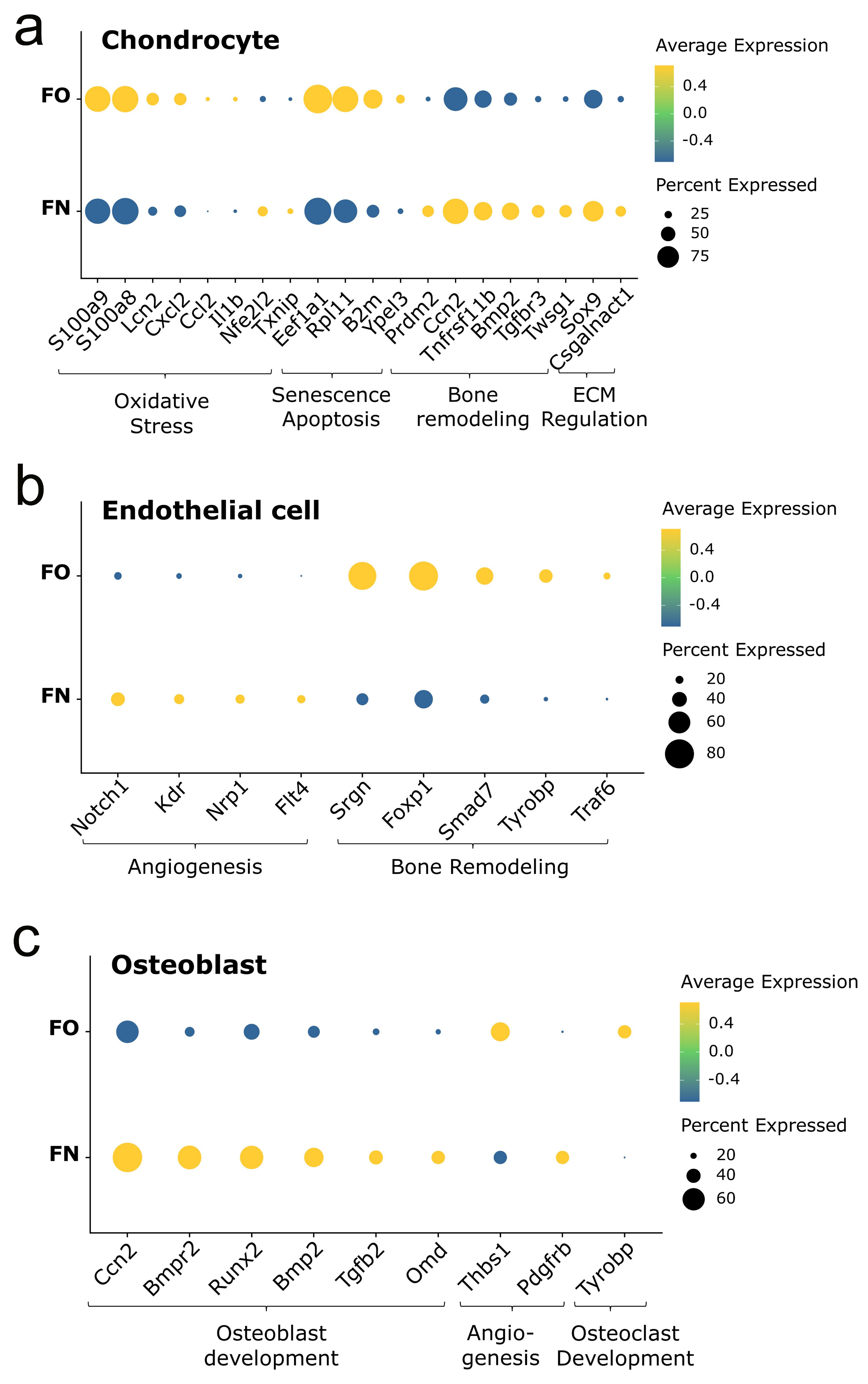


**Figure. S15.** Dot plots indicate differentially expressed genes of chondrocytes **(a)**, endothelial cells **(b)** and osteoblasts **(c)** between FO and FN groups. Presented genes are derived from the results of interest in the enrichment analysis, mostly refer to degeneration of chondrocytes, angiogenesis and remodeling of subchondral bone.

**
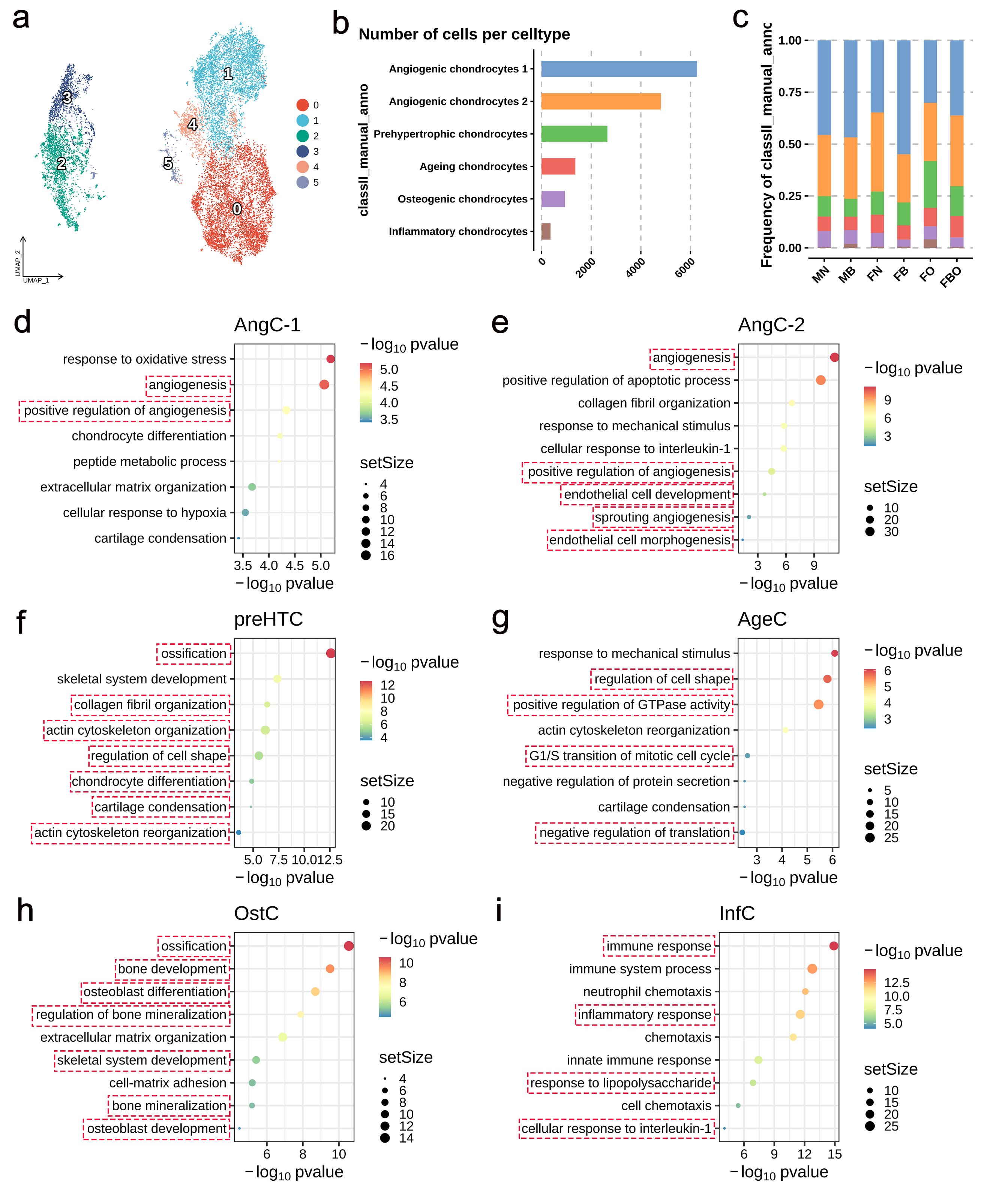
**

**Figure. S16.** ScRNA-seq atlas of chondrocytes subpopulations of femoral condyles for the six groups. **a** UMAP plot of chondrocyte single cells profiled in the presenting work colored by cell types. **b** Barplot shows the number of six sub types of chondrocytes. **c** Barplot shows the proportion of six sub types of chondrocytes in each group. **d** Functional enrichment analysis of highly expressed genes specific to AngC-1 using bubble plots, including angiogenesis, positive regulation of endothelial cell chemotaxis and so on. **e** Functional enrichment analysis of highly expressed genes specific to AngC-2 using bubble plots, including angiogenesis, positive regulation of endothelial cell proliferation and so on. **f** Functional enrichment analysis of highly expressed genes specific to preHTC using bubble plots, including ossification, actin cytoskeleton organization and so on. **g** Functional enrichment analysis of highly expressed genes specific to AgeC using bubble plots, including regulation of cell shape, positive regulation of GTPase activity and so on. **h** Functional enrichment analysis of highly expressed genes specific to OstC using bubble plots, including ossification, bone development and so on. **i** Functional enrichment analysis of highly expressed genes specific to InfC using bubble plots, including immune response, immune system process and so on.

**
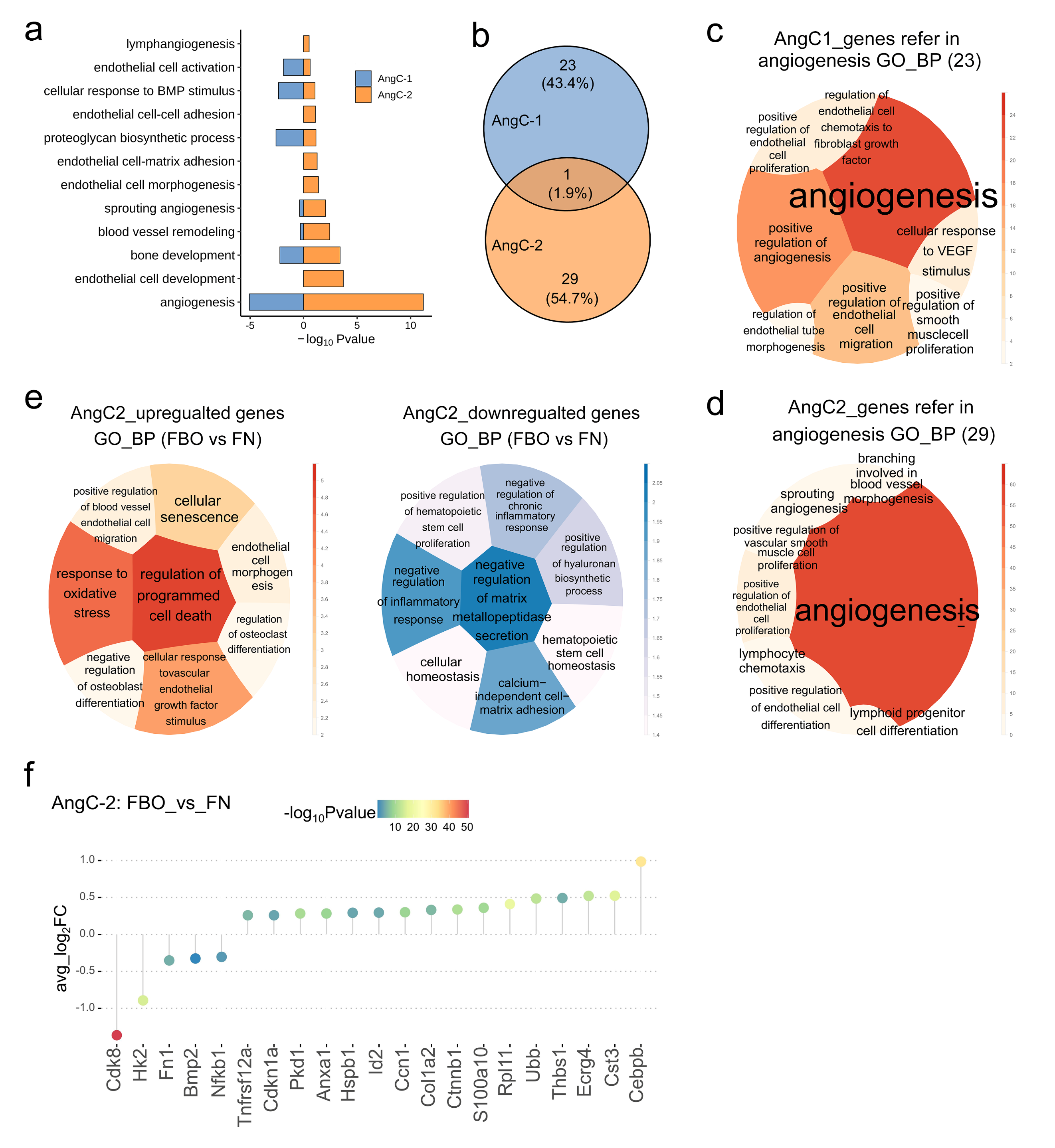
**

**Figure. S17.** Comparisons between AngC-1 and AngC-2. **a** The bar plot showed the specific biological functions of AngC-1 and AngC-2. The specific function is derived from the enrichment analysis of top genes in each sub cell types of chondrocytes calculated by “FindAllmarker”. The X-axis represents the -log_10_(*p*-value) of the pathway, and the length of the column indicates the significance of pathway enrichment in the cell types. **b** Venn plot shows the number of genes refer in the “angiogenesis” pathway to AngC-1 and AngC-2. **c** The red circle plot shows the enrichment results of 23 genes related with angiogenesis pathway in AngC-1. The deeper the color is, or the bigger the size is, the -log_10_(*p*-value) is greater, indicating a more significant difference. **d** The red circle plot shows the enrichment results of 29 genes linked to angiogenesis pathway in AngC-2. The deeper the color is, or the bigger the size is, the -log_10_(*p*-value) is greater, indicating a more significant difference. **e** The circle enrichment plot shows the GO database results of group FBO compared with group FN in AngC-2. The deeper the color is, or the bigger the size is, the -log_10_(*p*-value) is greater, indicating a more significant difference. Red (YlOrRd) circle plot indicates the up-regulation function of group FBO relative to group FN (left), and the blue (PuBu) circle plot indicates the down-regulation function (right). **f** Ggdotchart showed the expression of interest genes in group FBO and group FN in AngC-2. Genes are sorted from left to right according to their fold differences (avg_log_2_FC), with the vertical axis representing the magnitude of the fold differences. Genes with fold differences less than 0 are downregulated, while those with fold differences greater than 0 are upregulated. The color of the dot represents - log_10_ (P value), and the redder the color, the higher the significance.


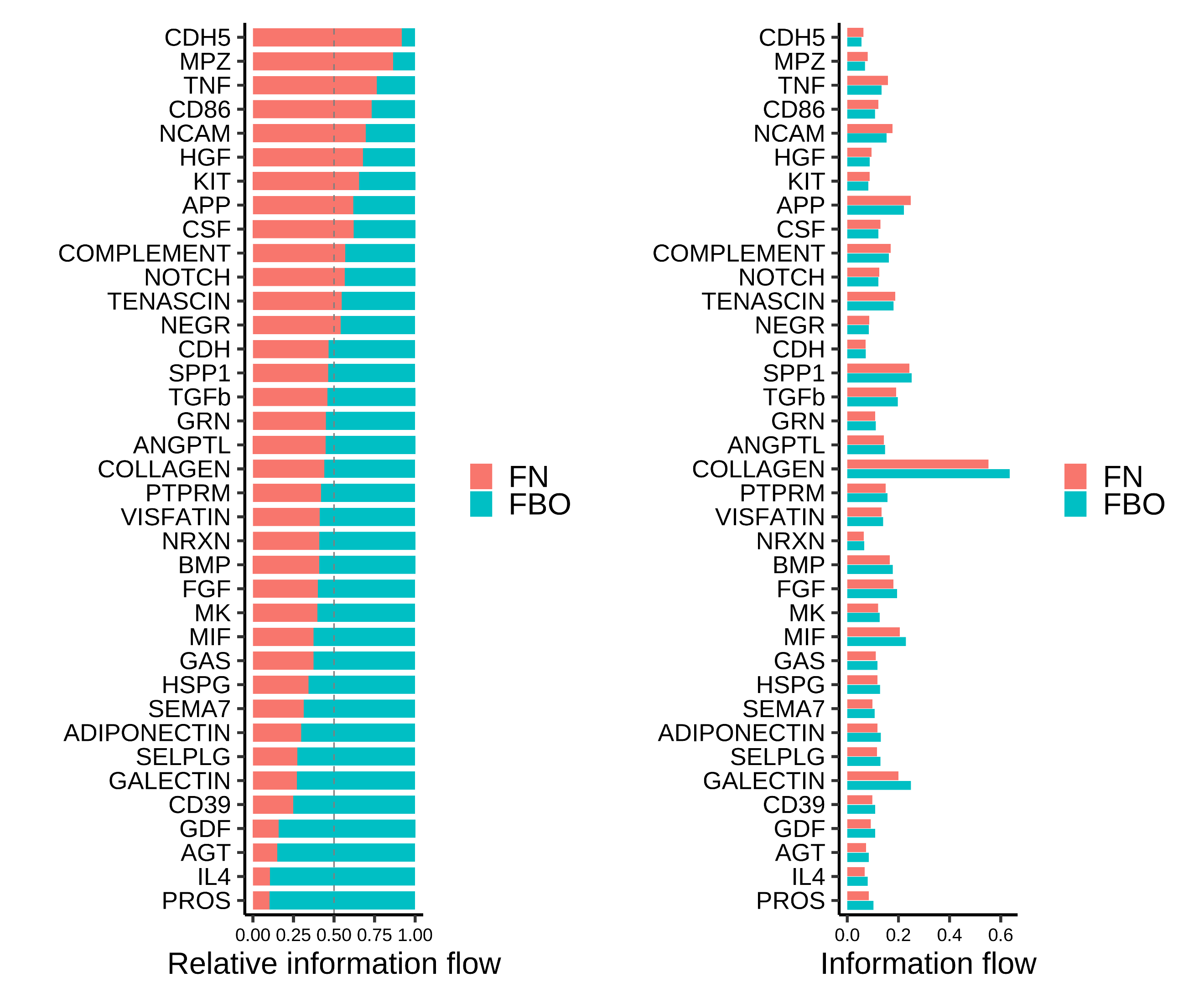


**Figure. S18.** The bar plot showed the strength of the interaction signal pathways for group FBO and group FN.


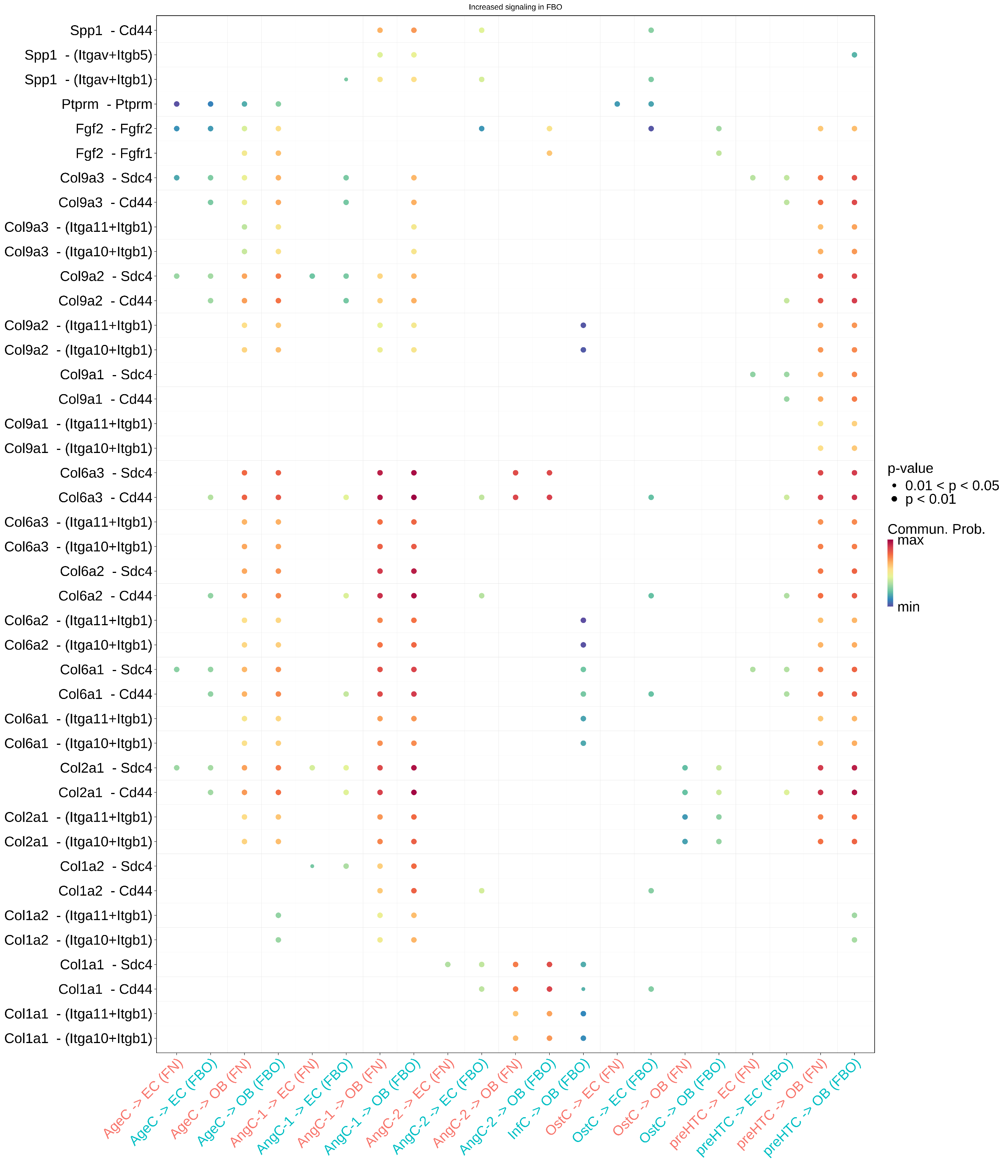


**Figure. S19.** Dotplot showed the signal pathway information of the interaction between interest cell types.

**
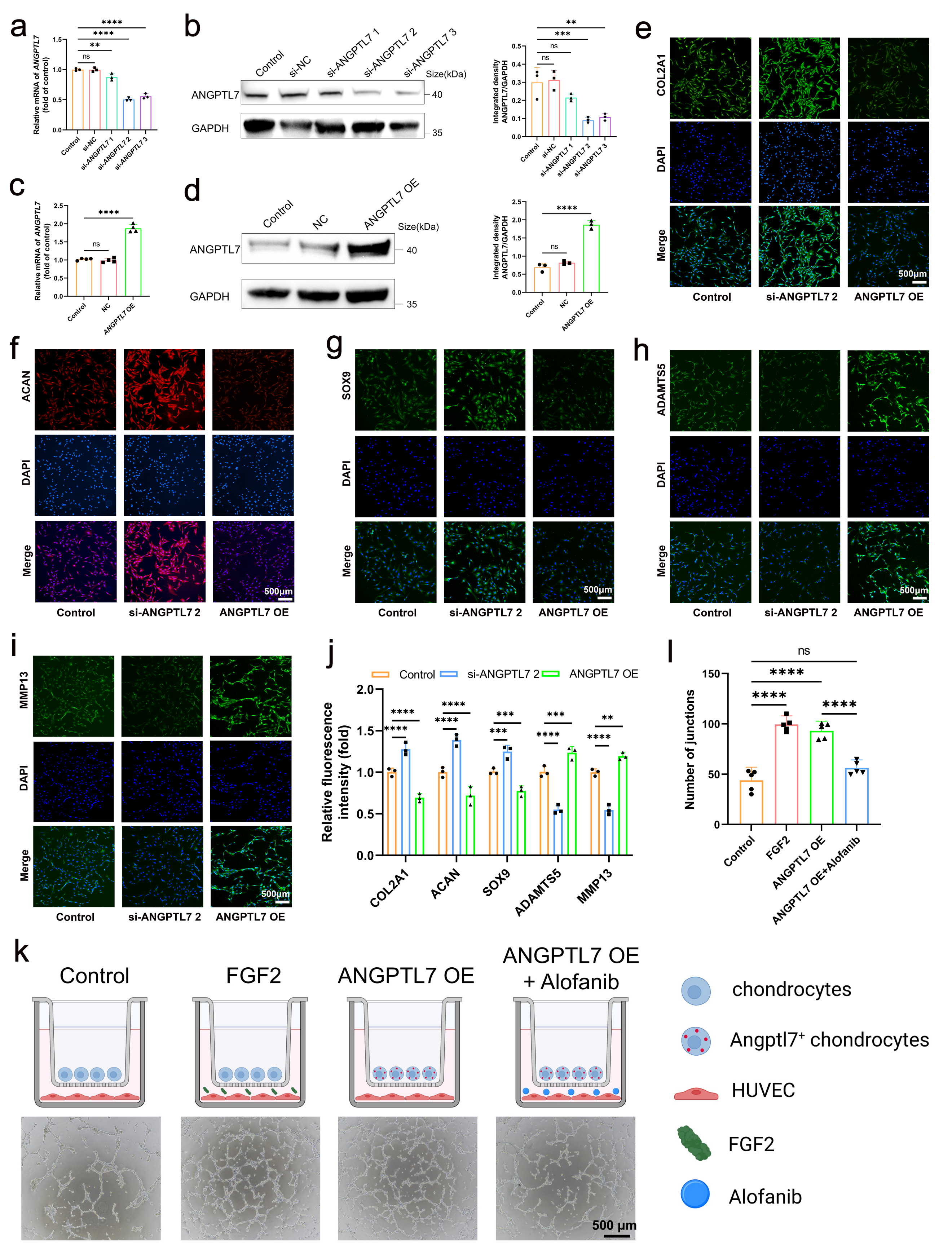
**

**Figure. S20.** Verification for the effects of *ANGPTL7*^+^ chondrocytes in KOA development. **a** Small interfering RNA (siRNA)-mediated knockdown of *ANGPTL7* gene expression in human chondrocytes was assessed using qPCR. **b** Western blot assay and semi-quantitative analysis of ANGPTL7 protein expression. **c** Lentivirus-mediated overexpression (OE) of *ANGPTL7* gene expression was assessed using qPCR. **d** Overexpression of ANGPTL7 was verified by the WB analysis. **e-i** Representative micrographs of immunofluorescence staining of COL2A1 **(e)**, ACAN **(f)**, SOX9 **(g)**, ADAMTS5 **(h)** and MMP13 **(i)** in human chondrocytes treated with PBS, si-ANGPTL7 2, and ANGPTL7-overexpressing lentivirus. Scale bar = 500 μm. **j** Semi-quantitative analysis of the fluorescence intensity of the tested proteins. **k** To verify that *ANGPTL7*^+^ chondrocytes promoted angiogenesis through the FGF2-FGFR2 signaling pathway, the tube formation assay was conducted using HUVEC cells. The HUVEC cells were co-cultured with the normal chondrocytes or *ANGPTL7*^+^ chondrocytes, respectively. And FGF2 and Alofanib (FGFR2 inhibitor) were added to the lower layer of HUVEC culture medium. Scale bar = 500 μm. **l** The number of junctions formed by the HUVECs under different treatments was quantitatively analyzed. Data are shown as mean ± SD, n = 3 per group. *P* values were determined by one-way ANOVA with a Tukey post hoc test for (**a-d, j** and **l**).


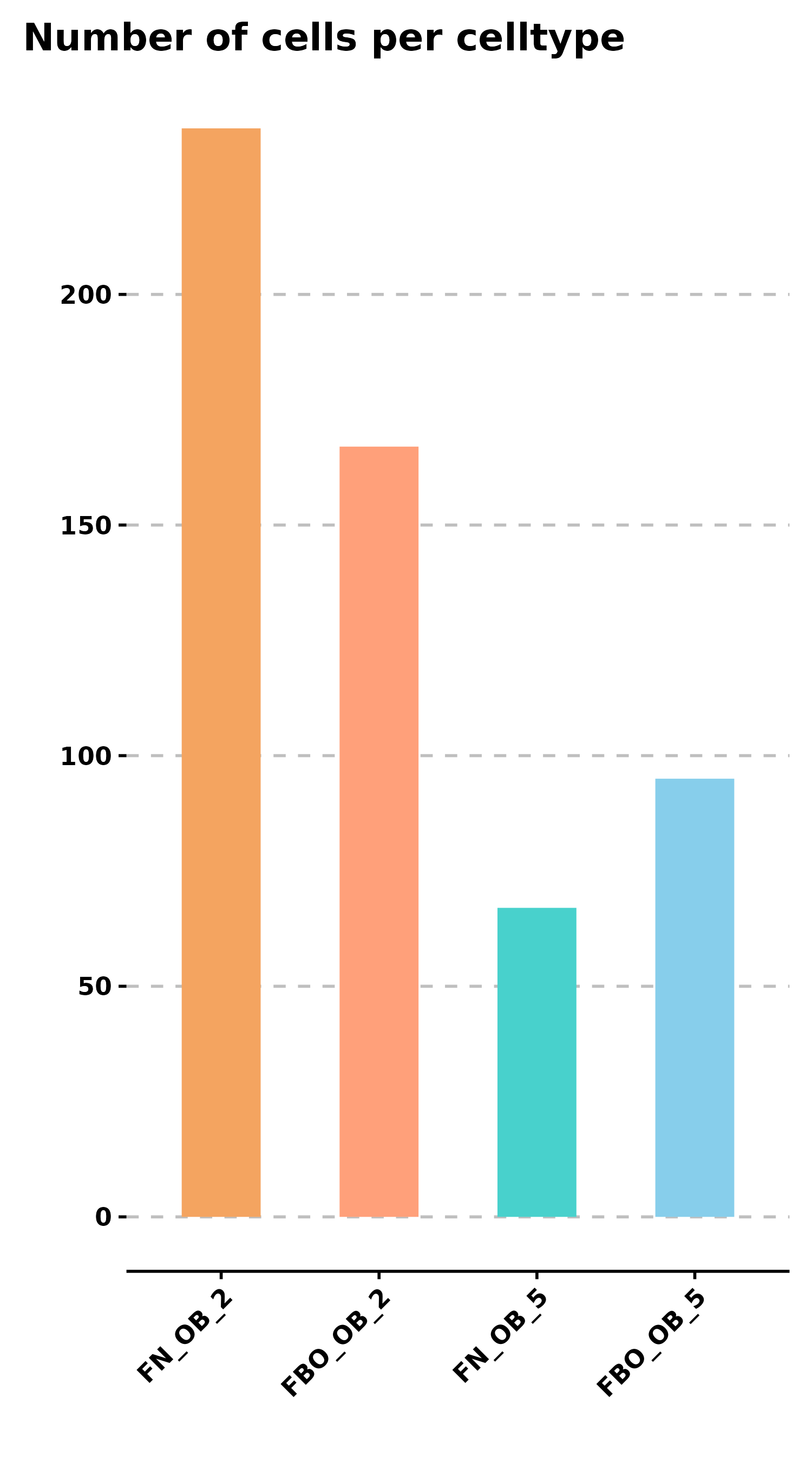


**Figure. S21.** Barplot shows the number of OB5 and OB2 in FN and FBO groups.

**
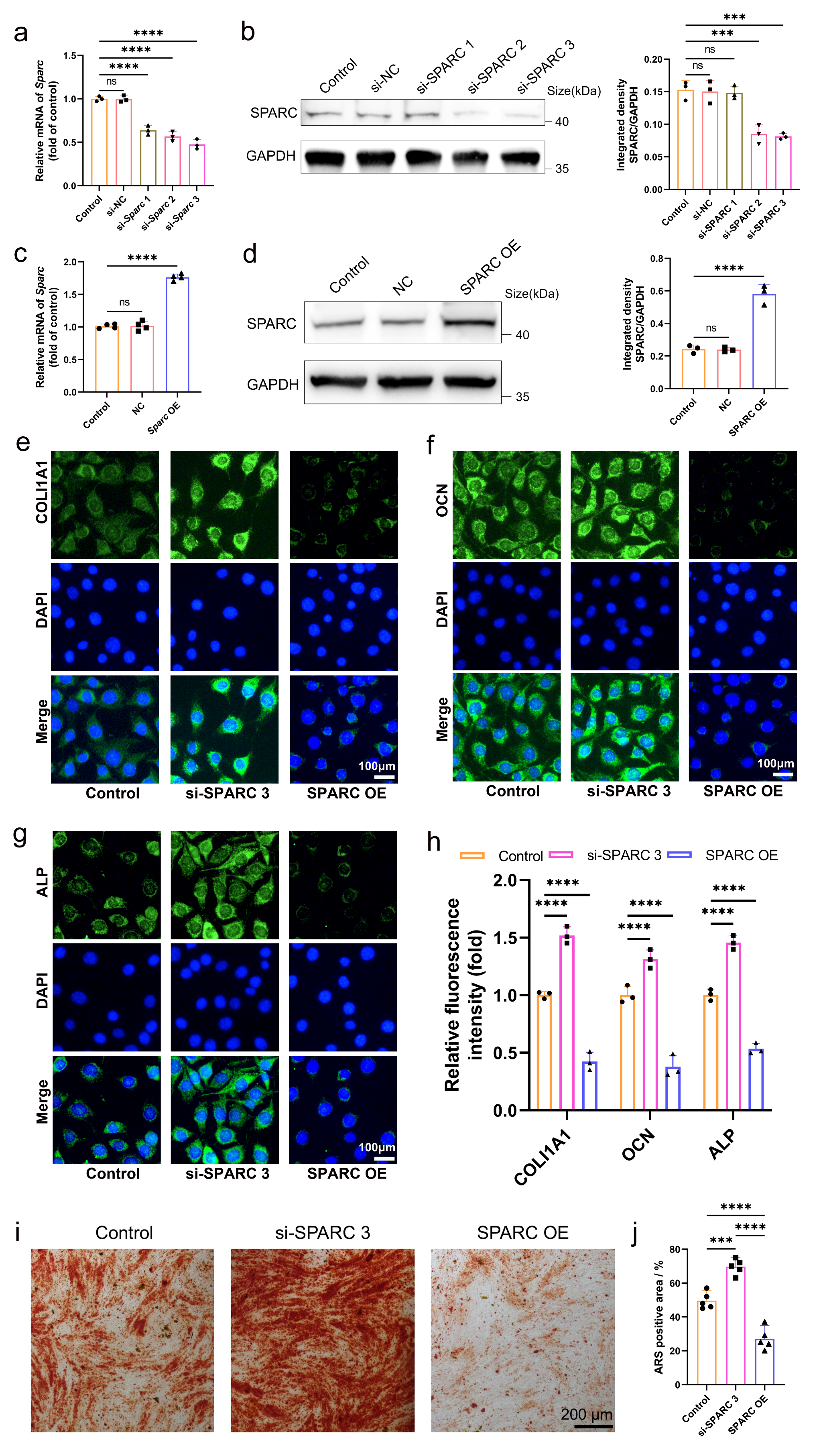
**

**Figure. S22.** Verification for the effects of *Sparc* gene in the osteoblast differentiation. **a** Small interfering RNA (siRNA)-mediated knockdown of *Sparc* gene expression in osteoblast-like *MC3T3* cells was assessed using qPCR. **b** Western blot assay and semi-quantitative analysis of SPARC protein expression. **c** Lentivirus-mediated overexpression (OE) of *Sparc* gene expression was assessed using qPCR. **d** Overexpression of SPARC was verified by the WB analysis. **e-g** Representative micrographs of immunofluorescence staining of COL1A1 **(e)**, OCN **(f)**, and ALP **(g)** in osteoblast-like *MC3T3* cells treated with PBS, si-SPARC 3, and SPARC-overexpressing lentivirus. Scale bar = 100 μm. **h** Semi-quantitative analysis of the fluorescence intensity of the tested proteins. **i** Alizarin Red staining (ARS) was performed for osteoblast-like *MC3T3* cells treated with PBS, si-SPARC 3, and SPARC-overexpressing lentivirus. Scale bar = 200 μm. **j** Semi-quantification for the ARS staining. Data are shown as mean ± SD, n = 3 per group. *P* values were determined by one-way ANOVA with a Tukey post hoc test for (**a-d, h** and **j**).


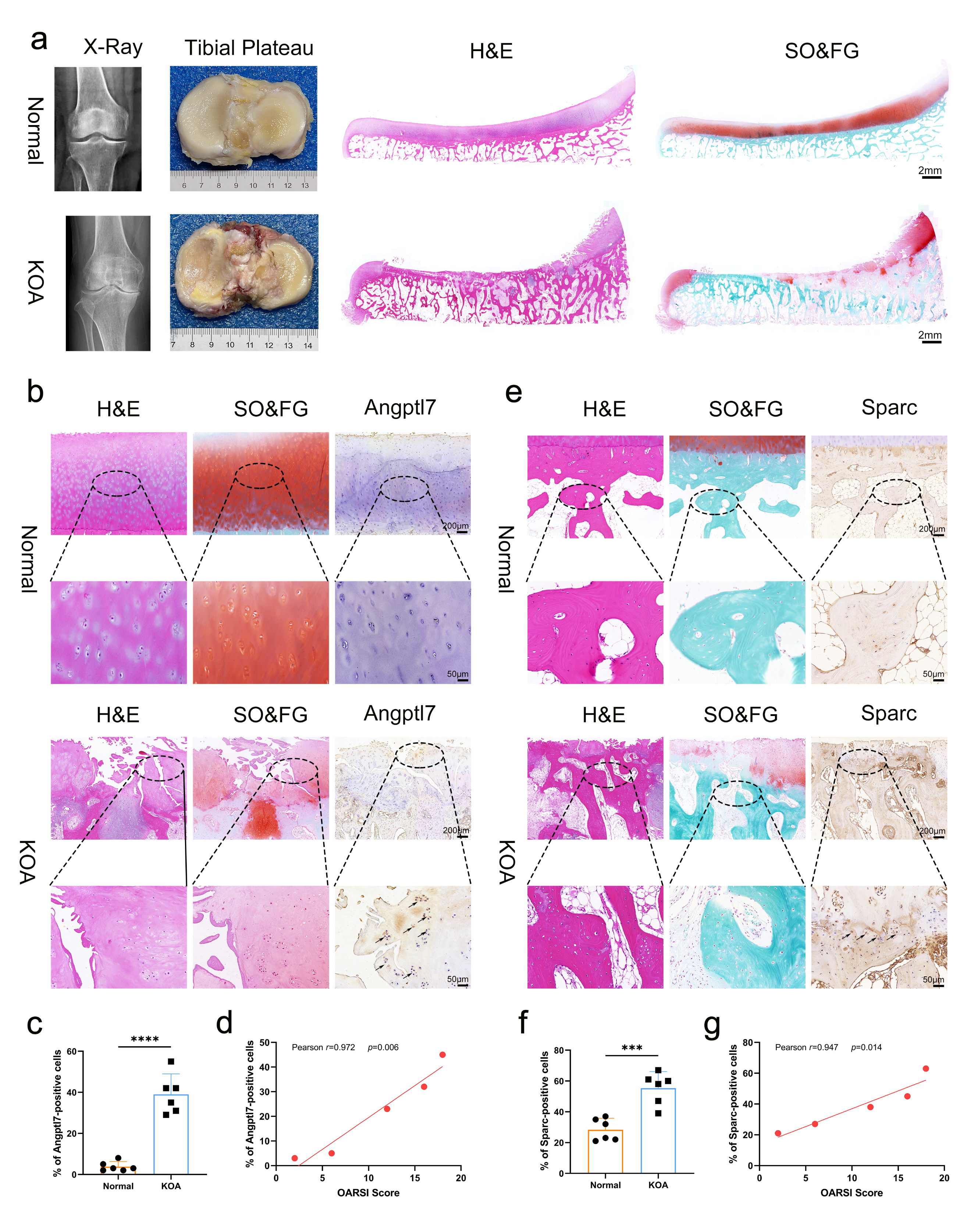


**Figure. S23.** Positive correlation between the expression level of *ANGPTL7* and *SPARC* genes with KOA progression. **a** Representative radiographic and macroscopic of tibial plateaus, and micrographs of H&E and SO&FG staining in coronal sections of tibial plateaus from the normal and KOA groups, Scale bar = 2 mm. **b** Representative micrographs of immunohistochemical staining of ANGPTL7 in coronal sections of the tibial plateaus in the normal and KOA groups, Scale bar = 200 μm, 50 μm. **c** Semi-quantitative analysis of immune-positive percentage of ANGPTL7 in tibial plateaus from the normal and KOA groups. **d** Correlation analysis between the percentage of ANGPTL7 immuno-positive cells and OARSI score in KOA patients. **e** Representative micrographs of immunohistochemical staining of SPARC in coronal sections of the tibial plateaus in the normal and KOA groups, Scale bar = 200 μm, 50 μm. **f** Semi-quantitative analysis of immune-positive percentage of SPARC in tibial plateaus from the normal and KOA groups. **g** Correlation analysis between the percentage of SPARC immuno-positive cells and OARSI score in KOA patients. Data are shown as mean ± SD, n = 3 per group. *P* values were determined by unpaired, two-tailed t-test for **c** and **f**, pearson correlation coefficients was performed by correlation analysis for **d** and **g.**

**Table S1 Clinical and demographic data of KOA patients included in this study**

| Patient | Age (years) | Gender | Side | CRP (mg/L) | ESR (mm/h) | HB (g/L) | ALB (g/L) | Bp  (mmHg) | Bs (mmoL/L) |
| --- | --- | --- | --- | --- | --- | --- | --- | --- | --- |
| 1# | 60 | F | R | 2.27 | 11 | 134 | 46.3 | 128/98 | 5.05 |
| 2# | 68 | F | R | 5.26 | 5 | 145 | 50.2 | 158/85 | 6.48 |
| 3# | 65 | M | L | 1.57 | 2 | 158 | 47.3 | 139/83 | 5.88 |
| 4# | 59 | M | R | 1.29 | 2 | 155 | 46.7 | 158/85 | 6.48 |
| 5# | 55 | F | L | 3.93 | 20 | 122 | 48.5 | 136/84 | 5.43 |
| 6# | 68 | M | L | 4.76 | 15 | 132 | 50.1 | 128/95 | 6.12 |
| 7# | 70 | F | R | 4.12 | 8 | 130 | 42.8 | 130/98 | 5.55 |
| 8# | 64 | F | R | 3.89 | 9 | 135 | 43.7 | 129/99 | 6.12 |
| 9# | 58 | M | L | 4.11 | 11 | 137 | 51.2 | 126/91 | 5.98 |
| 10# | 60 | F | R | 3.98 | 10 | 127 | 43.1 | 131/100 | 6.02 |

KOA: knee osteoarthritis; CRP: c-reactive protein; ESR: erythrocyte sedimentation rate; HB: hemoglobin; ALB: albumin; Bp: blood pressure; Bs: blood sugar. F: female; M: male; R: right; L: left.

**Table S2 Cell numbers before and after the quality control**

| Groups | Before QC | After QC | Keep (%) |
| --- | --- | --- | --- |
| FB | 13600 | 11250 | 82.72 |
| FBO | 12815 | 9548 | 74.51 |
| FN | 10806 | 8870 | 82.08 |
| FO | 27413 | 21809 | 79.56 |
| MB | 9606 | 7367 | 76.69 |
| MN | 7843 | 6647 | 84.75 |
| Total | 82083 | 65491 | 79.79 |

QC: quality control

**Table S3 Primer sequences of related genes for the qPCR*.***

| Genes | Forward sequence (5'→3') | Reverse sequence (5'→3') |
| --- | --- | --- |
| *GAPDH* | TCAAGATCATCAGCAATGCC | CGATACCAAAGTTGTCATGGA |
| *PIEZO1* | CAACGAGAAGCACATGCTG | ATTAGGGATGACCACAGACTG |
| *ADAMTS5* | GTTTACTCGGGAGGATTTATGTG | TCTGGAGAACATATGGTCCC |
| *MMP13* | CTGGGCCAAATTATGGAGGA | GAAACAAGTTGTAGCCTTTGGA |
| *COL2A1* | AGGAGACAGAGGAGAAGCT | CTTGAGGACCCTGGATTCC |
| *ACAN* | CTGACCAGACTGTCAGATACC | TCCTCACACCAGGAAACTC |
| *SOX9* | CTCTGGAGACTTCTGAACGA | ACTTGTAATCCGGGTGGTC |
| *ANGPTL7* | GCCCAAGTTGCCAACCTTAG | AGGGAAGAGCAGTCGTAGATG |
| *Sparc* | GTGGAAATGGGAGAATTTGAGGA | CTCACACACCTTGCCATGTTT |

**Table S4 Detailed information for the primary antibodies used in this study**

| Primary antibody | Brand | Catalog No. | Application |
| --- | --- | --- | --- |
| anti-TRAP | FUJIFILM Wako | 294-67001 | IHC-P |
| anti-OSTEOCALCIN | abcam | ab93876 | IHC-P |
| anti-PIEZO1 | ABclonal | A23380 | IHC-P |
| anti-PIEZO1 | HUABIO | M1005-2 | IF |
| anti-ADAMTS5 | HUABIO | ER1903 | IF |
| anti-MMP13 | HUABIO | ET1702 | IF |
| anti-COL2A1 | ABclonal | A1560 | IF |
| anti-ACAN | HUABIO | ET1704 | IF |
| anti-SOX9 | Invitrogen | 702016 | IF |
| anti-RPL11 | Invitrogen | PA5-27468 | IHC-P |
| anti-THBS1 | HUABIO | HA721916 | IHC-P |
| anti-CCN1 | Invitrogen | PA5-78022 | IHC-P |
| anti-COL1A1 | ABclonal | A22090 | IHC-P |
| anti-TIMP2 | ABclonal | A16439 | IHC-P |
| anti-CD31 | abcam | ab182981 | Multiplex IF |
| anti-EMCN | ABclonal | A22662 | Multiplex IF |
| anti-OSTERIX | abcam | ab209484 | Multiplex IF |
| anti-SMOC2 | Abiowell | AWA58230 | Multiplex IF |
| anti-ANGPTL7 | HUABIO | ER62667 | Multiplex IF |
| anti-COL9A3 | ABclonal | A9798 | Multiplex IF |
| anti-APOE | ABclonal | A0304 | Multiplex IF |
| anti-COL1A1 | ABclonal | A22090 | Multiplex IF |
| anti-CCL2 | ABclonal | A7277 | Multiplex IF |
| anti-FGF2 | ABclonal | A11488 | Multiplex IF |
| anti-FGFR2 | ABclonal | A2074 | Multiplex IF |
| anti-SPARC | ABclonal | A14494 | IHC-P |
| anti-ECM1 | ABclonal | A16368 | IHC-P |
| anti-MGP | ABclonal | A5439 | IHC-P |
| anti-SRGN | ABclonal | A6951 | IHC-P |
| anti-RUNX2 | HUABIO | ET1612-47-50 | IHC-P |
| anti-IBSP | Invitrogen | PA5-114915 | IHC-P |
| anti-ALPL | Invitrogen | MA5-24845 | IHC-P |
| anti-BMP2 | ABclonal | A0231 | IHC-P |
| anti-TNFRSF12A | HUABIO | ET1611-93 | IHC-P |
| anti-ECRG4 | HUABIO | ER63314 | IHC-P |
| anti-FGF2 | ABclonal | A11488 | IHC-P |
| anti-CYTL1 | Proteintech | 15856-1-AP | IHC-P |
| anti-ARHGEF12 | Proteintech | 22441-1-AP | IHC-P |
| anti-CAMP | Invitrogen | JJ2-072 | IHC-P |
| anti-COL10A1 | ABclonal | A13288 | IHC-P |
| anti-ANGPTL7 | Invitrogen | PA5-36575 | WB |
| anti-SPARC | ABclonal | A14494 | WB |

**Table S5 Key Resources**

| ENSEMBL Genome | “https://www.ensembl.org” |
| --- | --- |
| Gene Ontology | “http://geneontology.org” |
| KEGG | “https://www.kegg.jp” |
| STRING | “https://string-db.org” |
| Msigdb | “https://www.gsea-msigdb.org/gsea/msigdb/” |
| wikipath | “https://www.wikipathways.org/index.php/WikiPathways” |
| panther | “http://pantherdb.org” |

**Table S6 SiRNA sequences*.***

| SiRNA | 21nt guide (5′→3′) | 21nt passenger (5′→3′) |
| --- | --- | --- |
| siANGPTL7-1 | AAUAGUUCAUCCUGUUUACUG | GUAAACAGGAUGAACUAUUUA |
| siANGPTL7-2 | GAAGGUAGAUGGUGUUAUAGU | UAUAACACCAUCUACCUUCAG |
| siANGPTL7-3 | GAGACUCAAGCUUUGAGAAAG | UUCUCAAAGCUUGAGUCUCUG |
| siSparc-1 | UAAUGUAUUCAGUUAAAUCUA | GAUUUAACUGAAUACAUUAAC |
| siSparc-2 | GAGACUUUGAGAAGAACUACA | UAGUUCUUCUCAAAGUCUCGG |
| siSparc-3 | GGUGCUAACAUAGAUUUAACU | UUAAAUCUAUGUUAGCACCUU |

**Supplementary Video:**

**Movie S1. Activities of bipedal mice in feeding cage**

Following bilateral forelimb and tail amputation surgery, C57 mice received attentive care, including the provision of corn cob bedding conducive to the trauma model. Daily observation of bipedal exhibited normal behavior within the cage, displaying no difficulties with eating, drinking, or mobility.
